# The mutual benefits of comparing energy system models and integrated assessment models

**DOI:** 10.12688/openreseurope.15590.1

**Published:** 2023-05-02

**Authors:** Hauke Henke, Mark Dekker, Francesco Lombardi, Robert Pietzcker, Panagiotis Fragkos, Behnam Zakeri, Renato Rodrigues, Joanna Sitarz, Johannes Emmerling, Amir Fattahi, Francesco Dalla Longa, Igor Tatarewicz, Theofano Fotiou, Michał Lewarski, Daniel Huppmann, Kostas Kavvadias, Bob van der Zwaan, Will Usher

**Affiliations:** 1 Division of Energy Systems, KTH Royal Institute of Technology, Stockholm, 10044, Sweden; 2 Copernicus Institute of Sustainable Development, Utrecht Universiteit, Utrecht, The Netherlands; 3 Planbureau voor de Leefomgeving, Den Haag, The Netherlands; 4 Faculty of Technology, Policy and Management, Delft University of Technology, Delft, The Netherlands; 5 Potsdam Institute for Climate Impact Research, Potsdam, Germany; 6 E3-Modelling S.A., Panormou 70-72, Athens, Greece; 7 Energy, Climate, and Environment (ECE) Program, International Institute for Applied Systems Analysis (IIASA), Laxenburg, Austria; 8 Global Energy Systems Analysis, Technische Universität Berlin, Berlin, Germany; 9 RFF-CMCC European Institute for the Economics and the Environment (EIEE), Milan, Italy; 10 TNO Energy Transition, Amsterdam, The Netherlands; 11 The Institute of Environmental Protection – National Research Institute (IOS-PIB) / National Centre for Emissions Management (KOBiZE), Warsaw, Poland

**Keywords:** Net-zero scenario analysis, Climate change mitigation, Renewable energy transition, power system, open-source

## Abstract

**Background:** The transition to a carbon neutral society such as that envisaged in the European Union Green Deal requires careful and comprehensive planning. Integrated assessment models (IAMs) and energy system models (ESMs) are both commonly used for policy advice and in the process of policy design. In Europe, a vast landscape of these models has emerged and both kinds of models have been part of numerous model comparison and model linking exercises. However, IAMs and ESMs have rarely been compared or linked with one another.

**Methods:** This study conducts an explorative comparison and identifies possible flows of information between 11 of the integrated assessment and energy system models in the European Climate and Energy Modelling Forum. The study identifies and compares regional aggregations and commonly reported variables We define harmonised regions and a subset of shared result variables that enable the comparison of results across the models.

**Results:** The results highlight similarities and differences on final electricity demand, electricity supply and hydrogen across three levels of aggregation. However, the differences between the regional aggregation of the models limit detailed analysis.

**Conclusions:** This first-of-its-kind comparison and analysis of modelling results across model type boundaries provides modellers and policymakers with a better understanding of how to interpret both IAM and ESM results. It also highlights the need for community standards for region definitions and information about reported variables to facilitate future comparisons of this kind.

## 1 Introduction

Models of all kinds, scopes and goals are increasingly used in energy and climate policy advice and systems design at all scales, from global and regional down to national and sub-national scale. For example, integrated assessment models (IAMs) provide insights on the interactions between energy systems, the economy, land-use, and climate, increasingly needed for informing long-term policy making. Energy system models (ESMs), instead, provide in-depth and context-specific insights on the technological transition required to decarbonize the energy system with commonly more detailed representation of temporal and spatial details. For each of the two types of models, a body of literature has been built that compares and links complementary models. The comparison of models and their results commonly serves the purpose of better understanding the differences in the results of models, or of providing additional insights. The differences can be structural, i.e., in the set-up of the modelling framework, or parametric, which means related to the input data, the selected value of model parameters, and to the boundary conditions. Understanding the differences between models improves the understanding u of whether the insights derived from the models are robust or not. At the same time, comparisons allow identifying possible synergies and opportunities for model linking, in such a way for models to complement each other’s insights and provide enhanced information.

In the field of climate modelling, systematic model comparisons and the use of comparison metrics have already had a significant history.

The fields of integrated assessment modelling and energy systems modelling contain subjective assumptions to a higher degree than climate models. But despite the different nature of models there are potentially lessons to be learned from the experiences of the climate modelling community. In any case, in these two fields many comparisons have also been conducted among models of the same type, e.g., among IAMs ( Harmsen *et al.*, 2021) or ESMs ( Ruhnau *et al.*, 2022) separately. In some of these comparisons, metrics have been developed that allow a standardized comparison of models and results. Standardized comparison methods, in turn, allow repeatability and the expandability of comparison exercises, while also favouring the identification of common variables and indicators for model linking where models display the potential to provide complementary insights.

For IAMs, work has been conducted in recent years to systematically compare results across models using diagnostic indicators and diagnostic scenarios to verify the robustness of provided insights and to improve the understanding of differences in their results ( Harmsen *et al.*, 2021; Kriegler *et al.*, 2015). Like climate models and IAMs, ESMs are also commonly used to inform policy processes, particularly in decarbonisation efforts. Also for ESMs, model comparisons are a common practice, used to understand the differences in model results and derive robust insights across models which can be used for policy recommendations.

In contrast to the comparison of models, the linking of models connects two or more models with complementary capabilities. This can be done via a soft-link that keeps the models as independent systems that exchange variables and run iteratively until their solutions converge, or via a hard link that establishes a procedure that allows to run the models together. In the field of energy systems modelling a common link is between models that focus on capacity expansion and investment planning and models that focus on the operation of the modelled system. Linking such models increases the robustness of the results of the investment planning models ( Deane *et al.*, 2012). However, the linking can also involve a multitude of models with very different scopes. The H2020 project OpenENTRANCE developed an open modelling platform consisting of models and datasets that allow model linking and the investigation of the role of human behaviour in decarbonisation scenarios. The SENTINEL project, also a H2020 initiative, developed a platform that provides the possibility to select and link models, which when linked are suitable to answer specific questions related to decarbonisation.

Gardumi *et al.*, developed an integrated assessment framework by linking ten models of different kinds, with a pan-European ESM and a global computable general equilibrium model at the core, linking to models covering local to national aspects of society, environment, and the energy system. The developed framework provides insights beyond the energy system for ecosystems and society across multiple geographic scales, but does not involve any IAMs ( Gardumi *et al.*, 2022).

We can note that model comparisons are commonly within a group of the same model type, while model linking is often connecting models of different type. Until now there has been little effort to formalise the cross-comparison of model results across different model types, including IAMs and ESMs. To provide policymakers with more consistent messages, model comparisons among models of different type can contribute to a better understanding of the differences in results between these models, and the enhanced robustness of model-driven insights.

The model types that are compared in this paper, namely IAMs and ESMs, both apply quantitative methods to model the analysed systems. In the comparison we include the six IAMs:

- Integrated Model to Assess the Global Environment (IMAGE)
- MESSAGEix-GLOBIOM
- PROMETHEUS
- Regional model of investments and development (REMIND)
- TIAM-ECN
- World induced technical change hybrid model (WITCH)

And the five ESMs:

- Euro-Calliope
- Long-term investment model for the electricity sector (LIMES)
- The model for European energy system analysis (MEESA)
- The open source electricity model base for Europe (OSeMBE)
- The price-induced market equilibrium system (PRIMES)

IAMs model human-earth systems to generate insights into global environmental change and issues of sustainable development. Moreno *et al.* (2023) and Riahi *et al.* (2017) illustrate how IAMs are used to assess global pathways for the development of integrated resource systems and related greenhouse gas emissions. The latter developed the Shared Socioeconomic Pathways (SSPs), also using the models IMAGE, MESSAGE, REMIND, and WITCH. The TIAM-ECN model has been used to analyse the economic, societal and energy system implications of a hydrogen partnership between Europe and North Africa ( van der Zwaan *et al.*, 2021).

In contrast to IAMs, ESMs represent the energy system or sub-sectors of it, investigating the long-term technology deployment options and investment cycles or detailed system operation with the representation of individual countries or sub-national regions and temporal resolution of years to hours ( Pfenninger *et al.*, 2018). Tröndle (2020), for example, uses the ESM Euro-Calliope to investigate the trade-offs between using renewable energies locally or at the sites of the best resources and Pickering *et al.* (2022) use Euro-Calliope to identify near-optimal solutions for a decarbonised European energy system. The LIMES model, developed at the Potsdam Institute for Climate Impact Research (PIK), has been used to investigate the effect of the new EU Green Deal targets on the EU Emission Trading System ( Pietzcker *et al.*, 2021) and the interactions with the Market Stability Reserve ( Osorio *et al.*, 2021). Of the group of compared models, the two ESMs OSeMBE and MEESA have so far been least applied in the literature. OSeMBE is built using the open-source modelling framework OSeMOSYS ( Henke *et al.*, 2022). The MEESA model is based on OSeMOSYS as well, but uses a translation of the source code to GAMS and a modified set of equations ( Tatarewicz *et al.*, 2022).

In summary, the two model types differ in their scope and resolution, with IAMs providing global insights across a substantial proportion of the economy, but at a higher regional aggregation and a cruder temporal and technology resolution. However, the IAM PROMETHEUS and the ESM PRIMES have been repeatedly used to provide energy reference scenarios for the European Commission and thereby highlight that these two model types can complement each other.

The aim of this study is to describe the overlaps between integrated assessment and energy system models in the context of modelling possible European decarbonisation pathways and how these overlaps might vary depending on the model implementation. Comparing IAMs and ESMs at the same time, has the potential to bring about novel and urgently needed insights. For instance, in terms of the compatibility of long-term energy policies with the technical requirements of the energy system operation. Such comparisons across model types have been rarely realised, leading to a lack of agreement in terms of the viability of alternative energy transition strategies. Therefore, we want to focus here on the simultaneous comparison between IAMs and ESMs.

To achieve the aim of this study we follow three research questions. The first research question (RQ1) of methodological nature is if harmonized regions can be defined, and if there are commonly reported variables? We attempt to answer this research question in the next section identifying harmonised region aggregations and result parameters which allow the comparison of the IAMs and ESMs from the European Climate and Energy Modelling Forum (ECEMF) project included in this study. The second research question (RQ2) is what are the differences in the results of the compared models? And can they be related to structural reasons or parametric cause? With the mapping of regions and variables from the first research question, we try to answer the second research question by analysing the results of a diagnostic deep-decarbonisation scenario run by all the models. The third research question (RQ3) is how could the two assessed model types benefit from each other? To answer RQ3 we investigate the possibilities to exchange information across models, i.e., for what aspects can the models inform each other, for instance, with regards to different sectoral demands not covered by (some) ESMs, and rates of technology implementation over time and space.

## 2 Methodology

**Figure 1.**
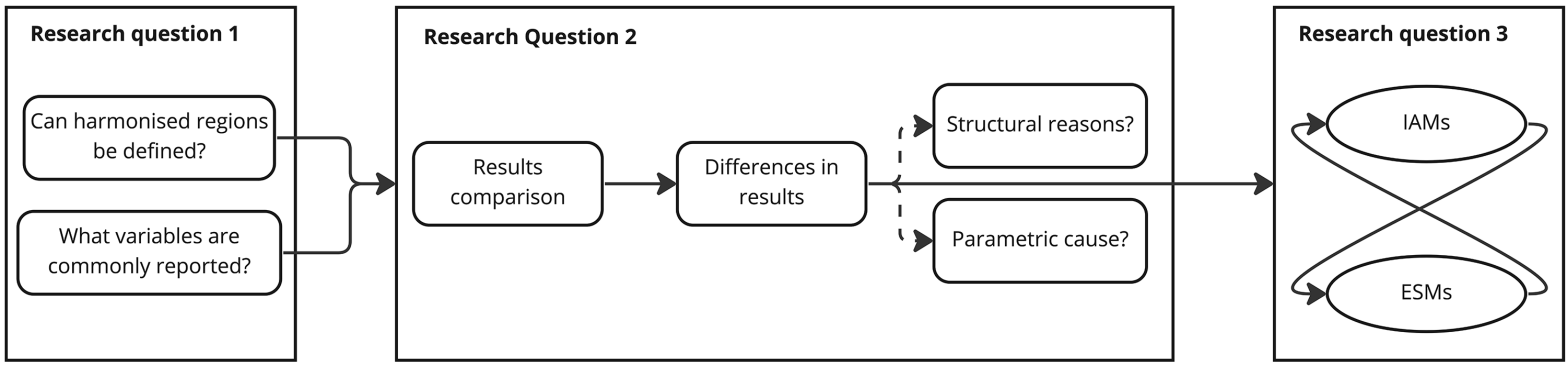
Linking research questions to methods.

In this section, we describe in detail the steps taken to meet the aim of the paper. In Section 2.1, we outline the selection criteria for including models in the study and describe the design of the diagnostic scenario used in this study. In Section 2.2, we describe the process by which we arrived at three levels of harmonised regions we can use to compare the model results. In Section 2.3 we describe the procedure used to identify common reporting variables. Figure 1 illustrates how the research questions are structured into sub-questions and steps, and how the sub-questions build up on each other to answer the overarching questions.

### 2.1 Selection of models for comparison

In the model comparison eleven IAMs and ESMs are compared. Table 1 provides an overview of the models, the compared version, the type, the regional aggregation of EU and UK, and a reference to their documentation.

**Table 1.** Integrated Assessment Models (IAMs) and Energy System Models (ESMs) in comparison exercise. Detailed descriptions of the IAMs and the option to compare their model design and logic are available at https://www.iamcdocumentation.eu/index.php/Model_comparison.

Abbr.	Model	Version	Type	Europe resolution	Documentation/website
EUR	Sector-Coupled Euro-Calliope	1.0	ESM	Country	( Pickering *et al.*, 2022)
IMA	IMAGE	3.2	IAM	2-region	https://models.pbl.nl/image/index.php/Welcome_to_IMAGE_3.2_ Documentation
LIM	LIMES	2.38	ESM	Country	https://www.pik-potsdam.de/en/institute/departments/transformation- pathways/models/limes
MEE	MEESA	1.1	ESM	9-region	( Tatarewicz *et al.*, 2022)
MES	MESSAGEix- GLOBIOM	1.2	IAM	2-region	https://docs.messageix.org/projects/global/en/latest/
OSE	OSeMBE	1.0	ESM	Country	https://osembe.readthedocs.io/en/latest/
PRI	PRIMES	2022	ESM	7-region	https://e3modelling.com/modelling-tools/primes/
PRO	PROMETHEUS	1.2	IAM	2-region	https://e3modelling.com/modelling-tools/prometheus/
REM	REMIND	2.1	IAM	9-region	( Baumstark *et al.*, 2021)
TIA	TIAM-ECN	1.2	IAM	2-region	https://www.iamcdocumentation.eu/index.php/TIAM-ECN
WIT	WITCH	5.1	IAM	18-region	https://www.witchmodel.org/

In the ECEMF project a set of diagnostic scenarios has been developed ( Dekker *et al.*, 2023). These scenarios aim to bring models into extreme states to explore their behaviour. However, in this paper the goal is to explore the overlaps and potential for linking IAMs and ESMs. To do so we believe it is sufficient to analyse results of one scenario. We select a diagnostic scenario with a high carbon price called DIAG-C400-lin with the carbon price increasing to 400 US$ in 2040 and to 580 US$ in 2050. This implies that we are analysing a scenario with deep decarbonisation towards 2050, i.e., the direction EU policy makers are aiming for.

### 2.2 Mapping model regions to harmonised regions for comparison

For both model types, it is widespread practice to define native model regions. These native regions aggregate collections of countries for which results are reported by the models. Models use aggregation to reduce computational demands. Some models, such as PRIMES, are specified at more detailed regional aggregation than that which their results are available. Especially among ESMs, some models report at more detailed spatial granularity, e.g., at the country or sub-country scale in the EU. The aggregation of countries to native regions can happen with different objectives in mind which as we show later creates differences across models, even when presenting the same European resolution. However, model results can only be compared when harmonised regions are identified. We define the following rules to identify a model region:

- A model must define one or more regions consisting of one or more countries.
- A country can only appear in one region.

Harmonised regions are defined as regions that appear in two or more models that contain the same countries. It is important to note that two models may use the same name for their model regions, but the pattern of countries contained do not match. It was necessary in this study to relax the strict definition of “exact match” to “or with a significant number of the same countries”. The definition of harmonised regions is not an explicitly spatial approach, but a way to define common aggregations for model nodes that represent regions of countries or individual countries.

**Figure 2.**
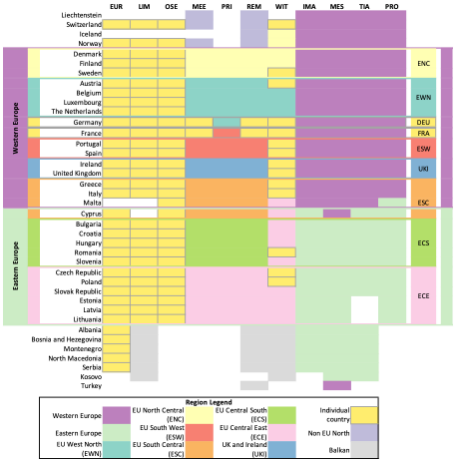
Region mapping for the EU27 & UK. The abbreviations used instead of model names are listed in Table 1.

**Figure 3.**
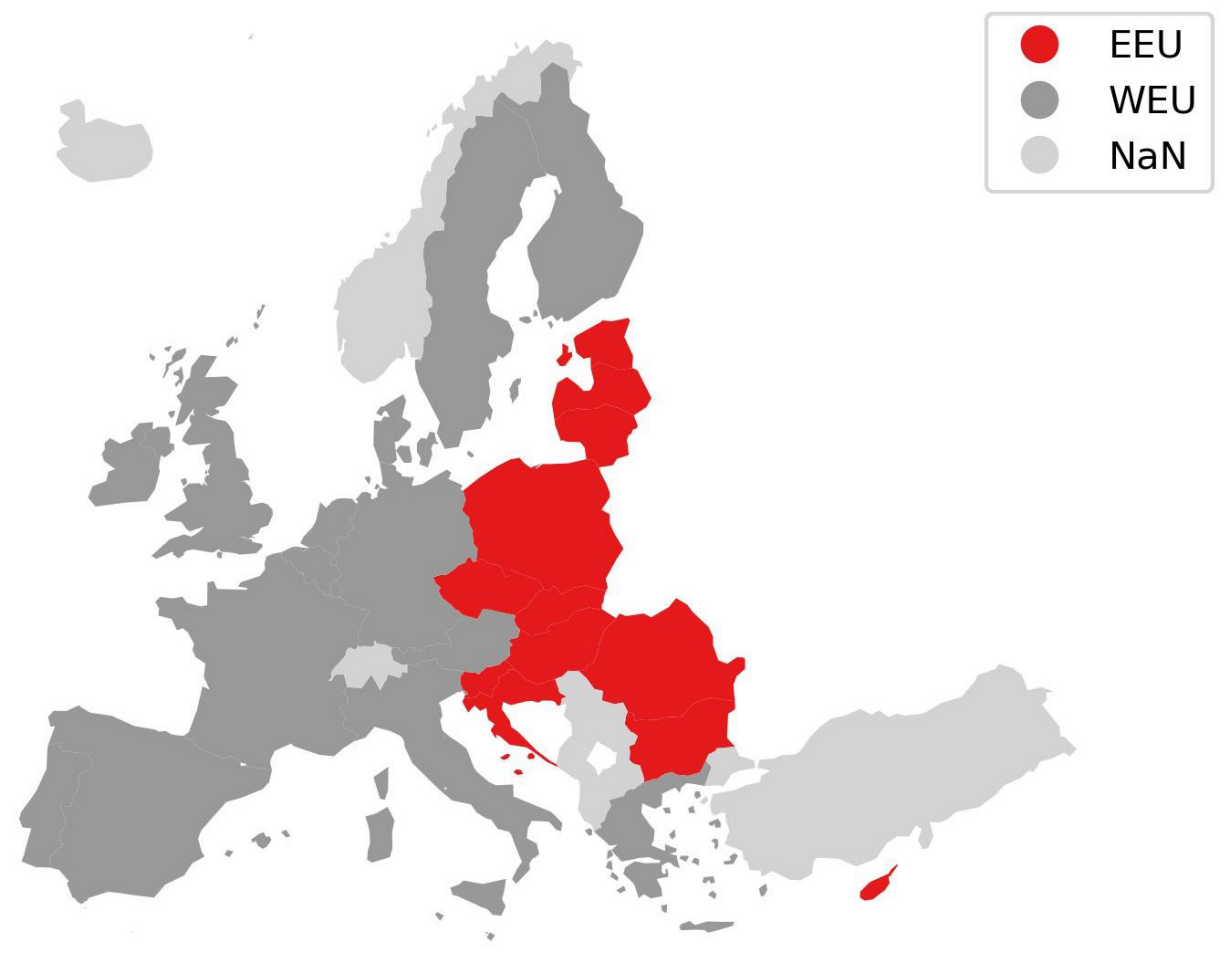
Harmonised two region mapping.

**Figure 4.**
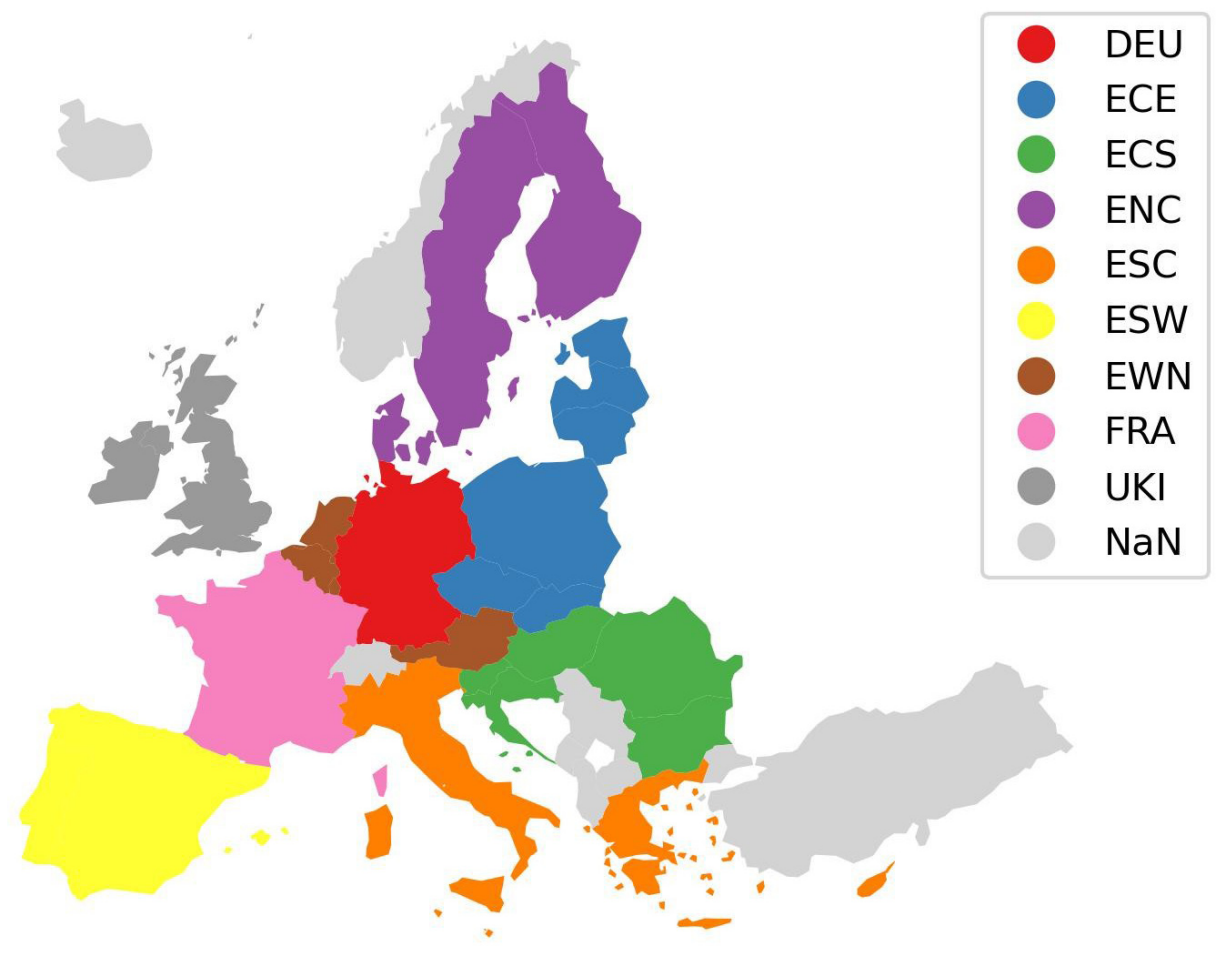
Harmonised nine region mapping.

To define harmonised regions for this paper, the first step was to collect the information on how the models involved in the comparison aggregate countries to native regions from model documentations (see Table 1 for references to documentation) and model mapping in the openENTRANCE Python package. In the second step the identified region aggregations are compared across models in tabular form and by visualising the region mapping. Lastly, based on this comparison harmonised regions are defined, that allow the comparison of as many models as possible at distinct levels of aggregation. The results of this process are documented comprehensively in Figure 2. The harmonised regions are additionally also shown in Figure 3 and Figure 4.

Figure 2 lists the models in the columns, and the countries in the rows. On the very left and right the derived harmonised regions are marked. Western and Eastern Europe are marked in purple and mint green. The horizontal lines between Norway and Denmark, Malta and Cyprus, and Lithuania and Albania mark the boarders of the harmonised regions of Western and Eastern Europe. The filled cells in the four columns on the right side of the figure for IMAGE, MESSAGEix-GLOBIOM, PROMETHEUS, and TIAM-ECN indicate which countries they consider part of Western and Eastern Europe. The two regions are to a large extent identical across the four models. The most significant difference is that MESSAGE-WEU includes Turkey, while the European regions of IMAGE, PROMETHEUS, and TIAM-ECN do not. Furthermore, the models vary in allocating the island countries Malta and Cyprus, which however are small and have a limited contribution in EU-wide pathways.

The second kind of aggregation is visible when looking at MEESA, PRIMES, and REMIND in the central columns of the figure. The most significant difference here is that MEESA and REMIND represent France and Germany as one-country-region each, while PRIMES integrates France and Germany respectively into the regions EU South West (ESW) and EU West North (EWN). MEESA and REMIND aggregate the remaining countries of the EU and UK into seven regions of two to six countries. This country aggregation was first implemented in the REMIND model by Rodrigues *et al.* (2022). Beyond the EU and UK, MEESA and REMIND also model and report results for “Non-EU North,” the Balkan countries, and Turkey; since they are outside the EU and UK region, they are not considered in this model comparison.

Thirdly, the mapping exercise in Figure 2 illustrates the approach taken by the WITCH model. In WITCH, 13 countries within the EU27 and the UK are modelled as single country regions (marked in yellow with a grey frame in Figure 2), while the remaining fifteen countries are aggregated into three regions with EU member states. Furthermore, Switzerland is modelled as a single country and the Balkan countries as one region.

Lastly, on the left side in Figure 2 are the models Euro-Calliope, LIMES, and OSeMBE. These three models do not aggregate the modelled countries to regions, but model each of the European countries individually or even at sub-national level in the case of Euro-Calliope (not shown in Figure 2).

In summary, the region mapping illustrates that there are four kind of resolutions that are used to model the EU and UK in the IAMs and ESMs involved in this comparison, namely aggregating countries into two regions (commonly Western and Eastern EU), grouping them into nine regions, grouping smaller countries into four regions and modelling the rest individually, and modelling countries individually. However, it is notable that models tend to vary in the allocation of countries. This can limit the comparability of results across models.

For the comparison in this paper, we derive two harmonised region aggregations which are marked in Figure 2. Figure 3 shows the harmonised two region aggregation for the EU and UK used in this paper. Cyprus is considered part of Eastern Europe and Malta is considered part of Western Europe, both cases follow the majority of “two-region” models compared. Turkey is not considered for the comparison.

Figure 4 shows the second harmonised region aggregation derived based on the aggregations used by the models MEESA, PRIMES, and REMIND aggregating the EU and the UK into nine regions. In the harmonised region aggregations the following regions exist:

- Harmonised two-region aggregation
  - Western Europe
  - Eastern Europe
- Harmonised nine-region aggregation
  - EU West North (EWN)
  - EU North Central (ENC)
  - EU South West (ESW)
  - EU South Central (ESC)
  - EU Central South (ECS)
  - EU Central East (ECE)
  - Germany (DEU)
  - France (FRA)
  - UK and Ireland (UKI)

An alternative approach to the manual mapping conducted here would be to use an explicitly spatial approach, mapping native regions to polygons representing the areas covered. Where differences, such as an overlap, are identified in aggregate regions between models, a spatial join or interpolation based on proxy variables (such as GDP and population for final energy demand) could be used to extract results for the individual country. However, this would be a much more labour-intensive approach and introduces considerable uncertainty and methodological complexity into the process of comparing results. In this first of its kind analysis, we limited the comparison to the presented harmonised regions.

### 2.3 Identifying common reporting variables

In parallel to identifying harmonised regions, we embarked on an investigation of common variables across both ESMs and IAMs. The reporting standard in the ECEMF project follows the IAMC-format, defined in the community-wide used database managed by IIASA and extensively used in many model intercomparison projects and in IPCC AR6 ( Huppmann *et al.*, 2021). In total, there are over 1,000 variables defined in the IAMC template, but only a subset of these are relevant to this study. The variables in IAMC-format can be both model inputs or outputs depending upon the model, and a variable that is an output for one model can be an input for another model.

The process of identifying common variables is manual, and was performed by examining the uploaded scenario data provided for the diagnostic scenario. However, modelling teams are continually updating their reporting of variables, and may add or remove variables over time. As such, the variables reported here are not necessarily representative of all the outputs available from the included models ( Cherp *et al.*, 2021).

Table 2 shows a list of identified common IAMC-format variables that are reported by IAMs and ESMs in the ECEMF project. They are selected to explain the most important aspects of the full energy (supply and demand) system.

**Table 2.** Variable mapping. The abbreviations for the model names are listed in Table 1.

	ESMs					IAMs					
Variable	EUR	LIM	MEE	OSE	PRI	IMA	MES	REM	PRO	TIA	WIT
**Capacity**											
Electricity|**	No	Yes	No	Yes	No	No	No	No	No	No	No
**Emissions**											
CO2|Energy|Supply|Electricity	No	Yes	Yes	Yes	Yes	Yes	Yes	Yes	Yes	Yes	Yes
CH4|Energy|Supply	No	No	No	No	Yes	Yes	No	Yes	No	Yes	Yes
Kyoto Gases|Energy	No	No	No	No	Yes	No	No	Yes	No	No	No
**Demands (Final Energy)**											
Final Energy	Yes	No	No	No	Yes	Yes	Yes	Yes	Yes	Yes	Yes
Electricity	Yes	No	No	Yes	Yes	Yes	Yes	Yes	Yes	Yes	Yes
Residential and Commercial	No	No	No	No	No	Yes	Yes	Yes	Yes	Yes	Yes
Residential and Commercial|Electricity	No	No	No	No	No	Yes	Yes	Yes	Yes	Yes	Yes
Commercial	No	No	No	No	Yes	Yes	No	No	No	Yes	Yes
Commercial|Electricity	No	No	No	No	No	Yes	No	No	No	Yes	Yes
Residential	No	No	No	No	No	Yes	No	No	No	Yes	Yes
Residential|Electricity	No	No	No	No	No	Yes	No	No	No	Yes	Yes
Transportation	Yes	No	No	No	Yes	Yes	Yes	Yes	Yes	Yes	Yes
Transportation|Electricity	Yes	No	No	No	Yes	Yes	Yes	Yes	Yes	Yes	Yes
**Primary Energy**											
Biomass|Electricity	No	Yes	Yes	Yes	Yes	Yes	No	Yes	No	No	No
Coal|Electricity	No	Yes	Yes	Yes	Yes	Yes	No	Yes	No	No	No
Gas|Electricity	No	Yes	Yes	Yes	Yes	Yes	No	Yes	No	No	No
Oil|Electricity	No	Yes	Yes	No	Yes	Yes	No	No	No	No	Yes
**Electricity Supply (Secondary** ** Energy|Electricity)**									
Biomass	Yes	Yes	Yes	Yes	Yes	No	Yes	Yes	Yes	Yes	Yes
Coal	Yes	Yes	Yes	Yes	Yes	Yes	Yes	Yes	Yes	Yes	Yes
Gas	Yes	Yes	Yes	Yes	Yes	Yes	Yes	Yes	Yes	Yes	Yes
Geothermal	No	No	No	Yes	Yes	Yes	Yes	Yes	No	Yes	No
Hydro	Yes	Yes	Yes	Yes	Yes	Yes	Yes	Yes	Yes	Yes	Yes
Nuclear	Yes	Yes	Yes	Yes	Yes	Yes	Yes	Yes	Yes	Yes	Yes
Ocean	No	No	No	Yes	Yes	No	No	No	No	No	No
Oil	No	Yes	Yes	Yes	Yes	Yes	Yes	Yes	Yes	Yes	Yes
Solar	Yes	Yes	Yes	Yes	Yes	Yes	Yes	Yes	Yes	Yes	Yes
Solar|CSP	No	Yes	No	No	Yes	Yes	Yes	Yes	No	Yes	Yes
Solar|PV	Yes	Yes	Yes	Yes	Yes	Yes	Yes	Yes	No	Yes	Yes
Wind	Yes	Yes	Yes	Yes	Yes	Yes	Yes	Yes	Yes	Yes	Yes
Wind|Offshore	Yes	Yes	Yes	Yes	Yes	Yes	Yes	Yes	Yes	Yes	Yes
Wind|Onshore	Yes	Yes	Yes	Yes	Yes	Yes	Yes	Yes	Yes	Yes	Yes
**Heat**											
Final Energy|Heat	No	No	No	No	Yes	Yes	Yes	Yes	Yes	Yes	No
Secondary Energy|Heat	Yes	No	No	No	Yes	Yes	Yes	Yes	No	Yes	No
**Hydrogen**											
Final Energy|Hydrogen	No	No	No	No	Yes	Yes	Yes	Yes	Yes	Yes	Yes
Secondary Energy|Hydrogen	Yes	No	No	No	Yes	Yes	Yes	Yes	No	Yes	Yes
Secondary Energy|Hydrogen|Electricity	Yes	Yes	Yes	No	Yes	Yes	Yes	Yes	No	Yes	Yes

The variable mapping determined that the power sector is the main set of IAMC variables that are shared by both IAMs and ESMs. Table 2 shows that there are few variables in the mapping that are not related to the power sector. The variables in the mapping can be grouped into seven categories: capacity, emissions, demands, primary energy, electricity supply, heat, and hydrogen.

Another insight Table 2 provides is the lower detail that ESMs provide for demand side variables. With the exception of PRIMES, none of the other ESMs report Final Energy for the residential and or commercial sector or Heat, and also final energy in transport is only reported by Euro-Calliope and PRIMES.

It is also notable that most ESMs do not fully cover hydrogen and heat, which is a challenge when investigating the synergies offered by sector coupling and possible scenarios arising from electrification, e.g., increasing use of heat pumps for heat generation.

## 3 Results

To investigate the second research question of the study, we created plots for each of the three harmonised region aggregations for the 11 models, for each of the common reporting variables.

The identified commonly reported variables are compared across models for the harmonised regions. At the lowest regional aggregation, only those models at country scale are compared. At medium aggregation, results for lower aggregations are summed (e.g. emissions) or averaged (prices), and models whose native regions match the harmonised regions are included. Where a mismatch occurs, the results are excluded for the harmonised region. At the highest aggregation, i.e., the two-region aggregation, all models are included in the comparison.

This section presents, compares, and analyses a selection of the above identified variables across models and geographic resolutions. Variables from the categories ‘demands’, ‘electricity supply’, and ‘hydrogen’ are presented and analysed. The demand side variables are selected since the demands drive the energy system development and therefore the demand levels have an important influence on the system design proposed by the model. In the variable category ‘electricity supply’ variables are selected for comparison that indicate the development of crucial low- and zero-emission power generation technologies. For this we compare the most mature low-emission technologies solar, wind, and nuclear. Across decarbonisation pathways, variable renewable energy (vRE) sources play a key role. To play this role and contribute to the decarbonisation of societies’ energy supply, decarbonisation pathways foresee a rapid growth of power generation from vREs in Europe over the next two decades.

Lastly, we compare the results for hydrogen. Even though not all models cover the entire energy system, most models cover hydrogen. But since hydrogen is expected to play a crucial role in decarbonising the economy by 2050, we consider a comparison of how the different models deal with hydrogen interesting.

The variables are plotted in three resolutions as analysed above with the EU and UK split into two regions, nine regions and country resolution.

There has not been an alignment of inputs across the models in the ECEMF project, but a review of their results when the same carbon price trajectory is imposed and in some cases updates of input data. The section first presents and analyses demand variables, followed by electricity supply variables, and lastly hydrogen.

### 3.1 Demands

**Figure 5.**
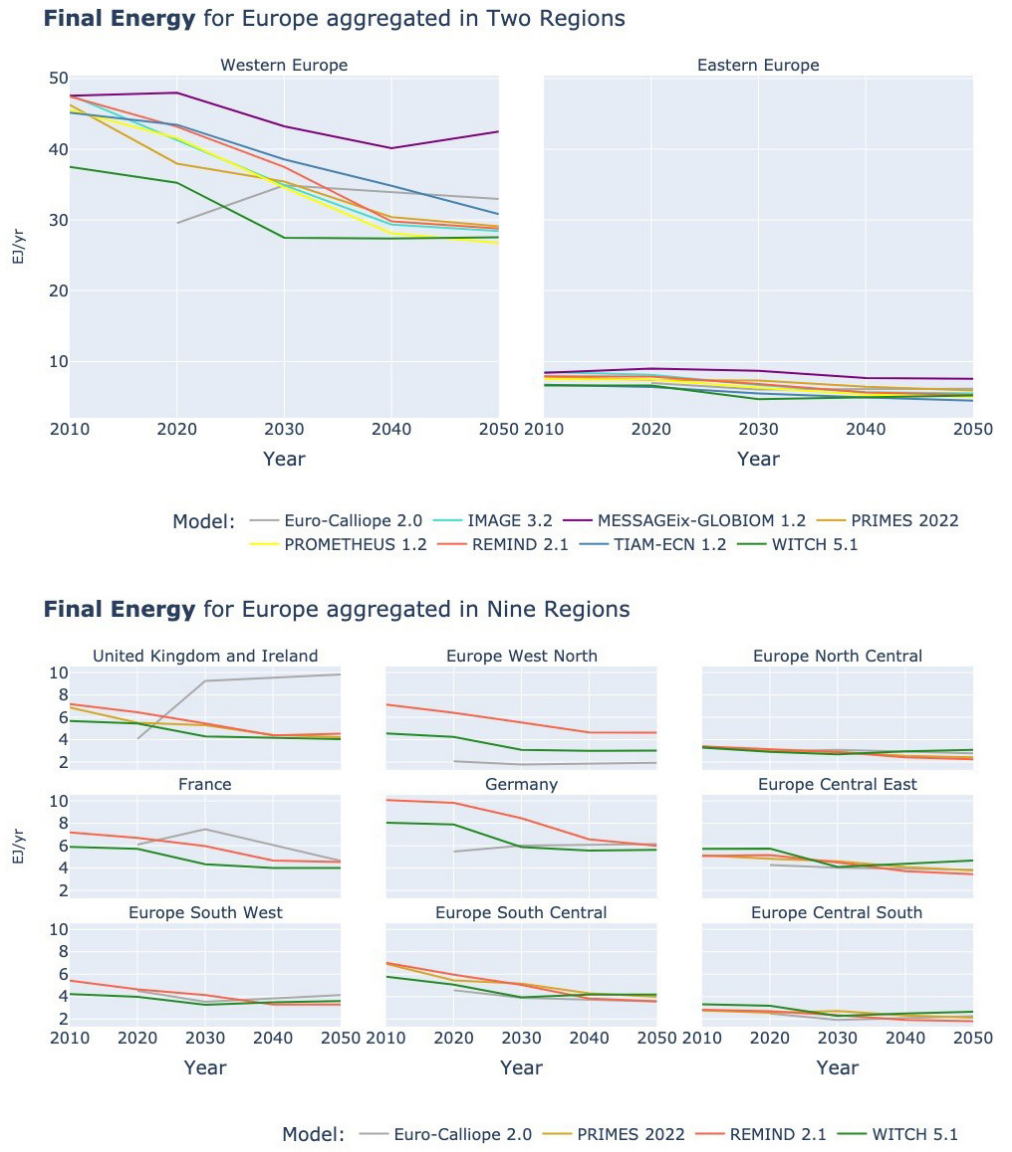
Final Energy at two resolutions across models.

The results for Final Energy show mostly a decrease. To 2030 all models show decreasing energy demand, but from 2030 and 2040 WITCH and MESSAGEix show increases again. All others keep decreasing, but at a slower pace. We can observe that these trends are not driven by any specific region (see Figure 5), and they are not caused by any specific demand sector (as observable in the extended data [ Henke, 2023b]). 

**Figure 6.**
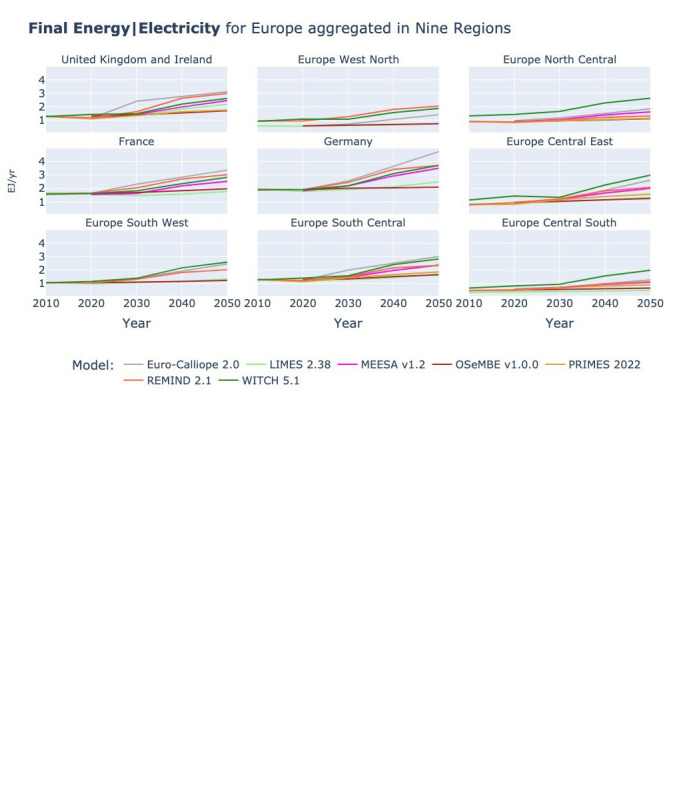
Final Energy Electricity at two resolutions across models.

In contrast to the slightly mixed picture for the overall Final Energy, Final Energy Electricity as shown in Figure 6 is consistently increasing across all models and regions, a result of the increasing electrification expected by all models with the rapid uptake of electric vehicles and heat pumps. We cannot observe a division between models of different types, even though in the supply side focused ESMs the electrification is a modeler’s decision whereas in the IAMs and the ESMs with more demand side detail the electrification is a model decision driven by the cost-competitiveness of different technologies and energy carriers to cover the demand by sector.

In the two-region plot, we can note that the above presented aggregation of countries into Western and Eastern Europe, with larger European countries being in Western Europe, leads to higher energy demand in Western Europe. However, the spread between models in final electricity demand in 2050 is almost identical. In Eastern Europe, the WITCH model has a demand that is 4.3 times higher than the lowest demand by TIAM-ECN and in Western Europe the demand in WITCH is 4.4 times higher than the lowest demand by IMAGE.

### 3.2 Electricity supply

**Figure 7.**
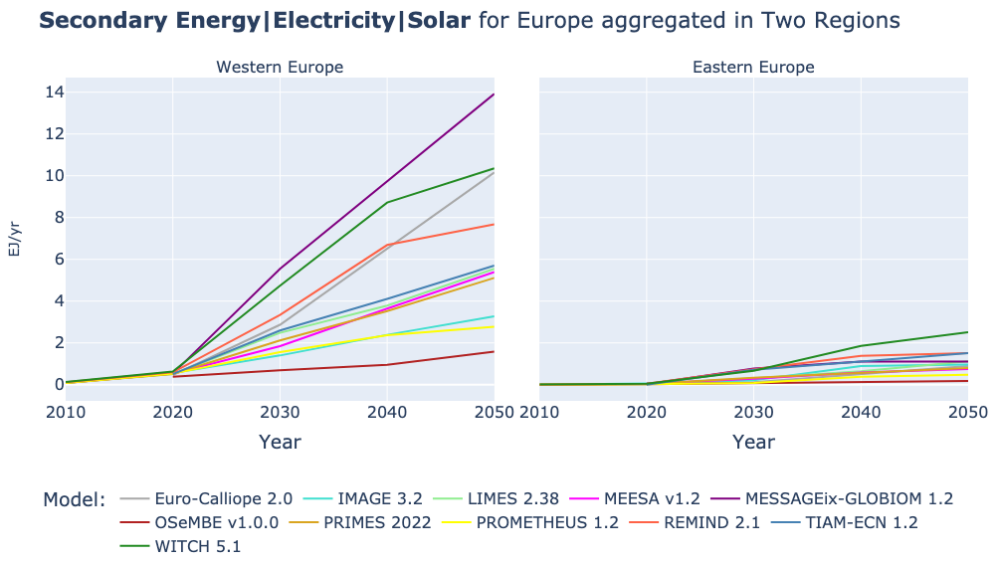
Secondary Energy Electricity Solar at two region aggregation across models. Please note, MESSAGEix-GLOBIOM counts Turkey as part of Western Europe.

***3.2.1 Solar power.*** All models present a strong growth in solar power generation over the period 2020-2050. It consists purely of solar PV power generation for most models, and for the models that report numbers on concentrating solar power (CSP) – see Table 2 – solar PV is the main driver of solar based power generation, see also Extended data ( Henke, 2023b). However, in Western Europe we can see a division between models that foresee a solar power generation in 2050 of 7.5-14 EJ/yr and models that are in a much lower range, between 1.6 and 5.5 EJ/yr, see Figure 7. When comparing the growth of solar power generation with the development of the final electricity demand in Figure 6 there is a correlation between models with a high solar power generation and a high final electricity demand. However, for final electricity demand the clear split into two groups of models in Western Europe for solar PV is not observed.

**Figure 8.**
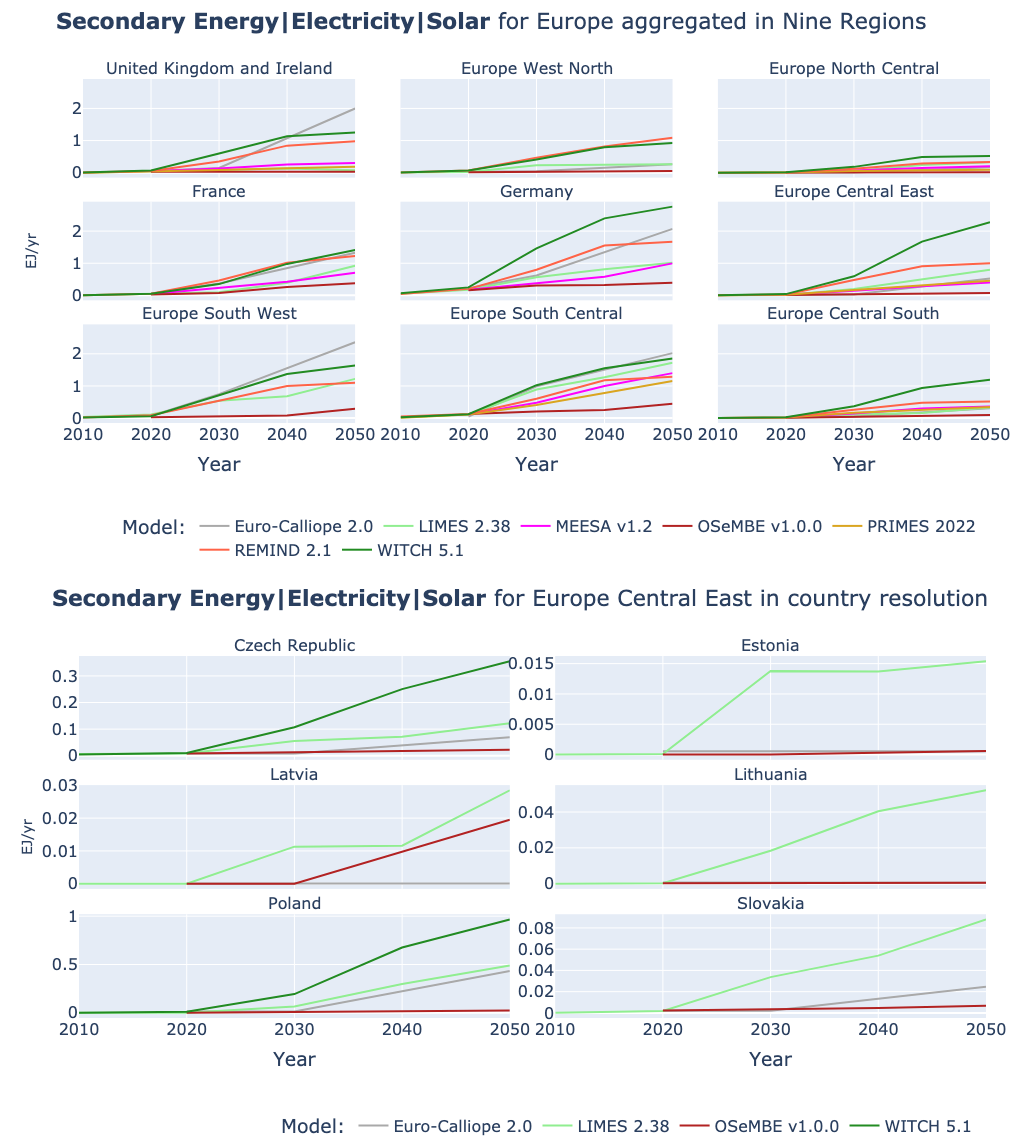
Secondary Energy Electricity Solar at nine region aggregation (above) and Europe Central East at country resolution (below).

Figure 8 shows in the upper half the development of solar power generation in Europe in the nine-region aggregation. The highest solar power generation is in Germany, France, and Europe South West (Spain and Portugal). In Europe Central East especially, we observe a variation in expected power generation across models. Therefore, the lower half of Figure 8 shows the countries that constitute Europe Central East. From the six models that are reporting results for Europe Central East only the three models (Euro-Calliope, LIMES, and WITCH) are also reporting at country level in the region. However, in the reported results we can note that the Poland contributes about half of the solar power generation in the region, followed by Czech Republic with only about 15% of the solar power generation in the region. Across the regions we can observe a repeating pattern across countries in which the WITCH model has the highest expected solar power generation followed by LIMES and Euro-Calliope, and OSeMBE with the lowest generation. OSeMBE generates more power from solar than Euro-Calliope in Latvia only. Even though it is consistent throughout the countries of Europe Central East, it is interesting to observe the lower power generation by solar in Euro-Calliope in comparison to the other models, because in most of the other eight regions (see upper half of Figure 8) Euro-Calliope belongs to the models with high expectations for solar power deployment.

**Figure 9.**
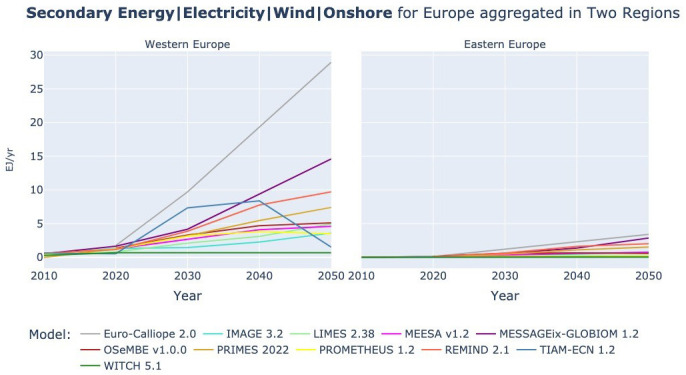
Secondary Energy Electricity Wind Onshore in two region aggregation across models.

**Figure 10.**
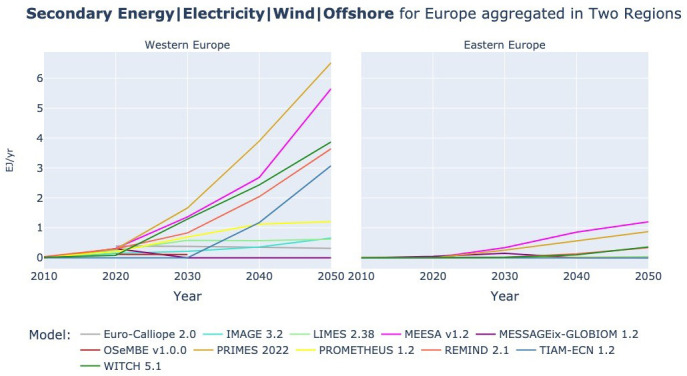
Secondary Energy Electricity Wind Offshore at two region aggregation across models.

***3.2.2 Wind power.*** Figure 9 and Figure 10 show the development of onshore and offshore wind power across models and regional aggregation. Euro-Calliope and WITCH produce most onshore wind power, MEESA and PRIMES produce most offshore wind power instead. While WITCH also has high electricity demand (see Figure 6), Euro-Calliope, PRIMES, and MEESA are rather in the middle of the electricity demand range. Hence, the differences in selection of wind power technology might not only follow the logic that, with higher demand levels, offshore wind is installed in addition to onshore wind. But this possibly indicates different modelling of wind resource limits: use of electricity for hydrogen production, e.g., Euro-Calliope (see Figure 13), use of different future cost estimates, or varying constraints in terms of integrability of wind into the power system. The plots for the lower regional aggregations are available in the extended data ( Henke, 2023b).

***3.2.3 Share of variable Renewable Energy Sources.*** In the sections on solar and wind power we highlighted the link between the amount of power generated by the two technologies and the final electricity demand and how this link can partly explain the differences in power generation across models. To eliminate the difference caused by the different electricity demand levels, this section analyses the variation of vRE shares in secondary electricity demand across models.

**Figure 11.**
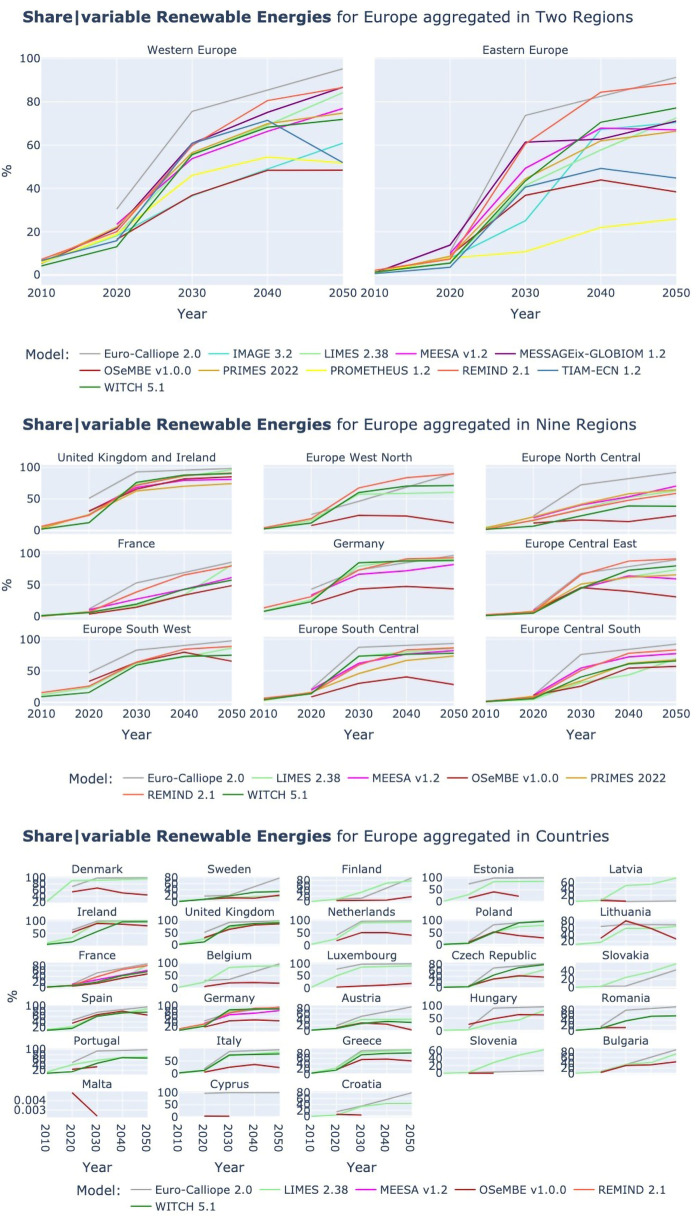
Share of variable Renewable Energy Sources at three aggregations across models. Two region aggregation at the top, nine region aggregation in the middle, and country resolution at the bottom.

As for solar and wind, there is a significant variation in the vRE share across models, see Figure 11. The spread in solar and wind power generation is not solely caused by the differences in electricity demand. For some models, model-specific patterns of high shares or low shares of vREs across regions and resolutions are observable, e.g., Euro-Calliope and REMIND are for most regions and countries on the upper end of the model spectrum and OSeMBE and PROMETHEUS are on the lower end. However, there are also models that vary in their position in comparison to the other models, e.g., the WITCH model shows the lowest vRE share in 2050 for the UK and Spain, while Poland is the highest.

***3.2.4 Nuclear power.*** As for the renewable energy, the deployment of nuclear power is to some extent related to demand developments. LIMES, IMAGE, and OSeMBE all have low final electricity demands (see Figure 6) and are also on low side regarding nuclear power generation.

**Figure 12.**
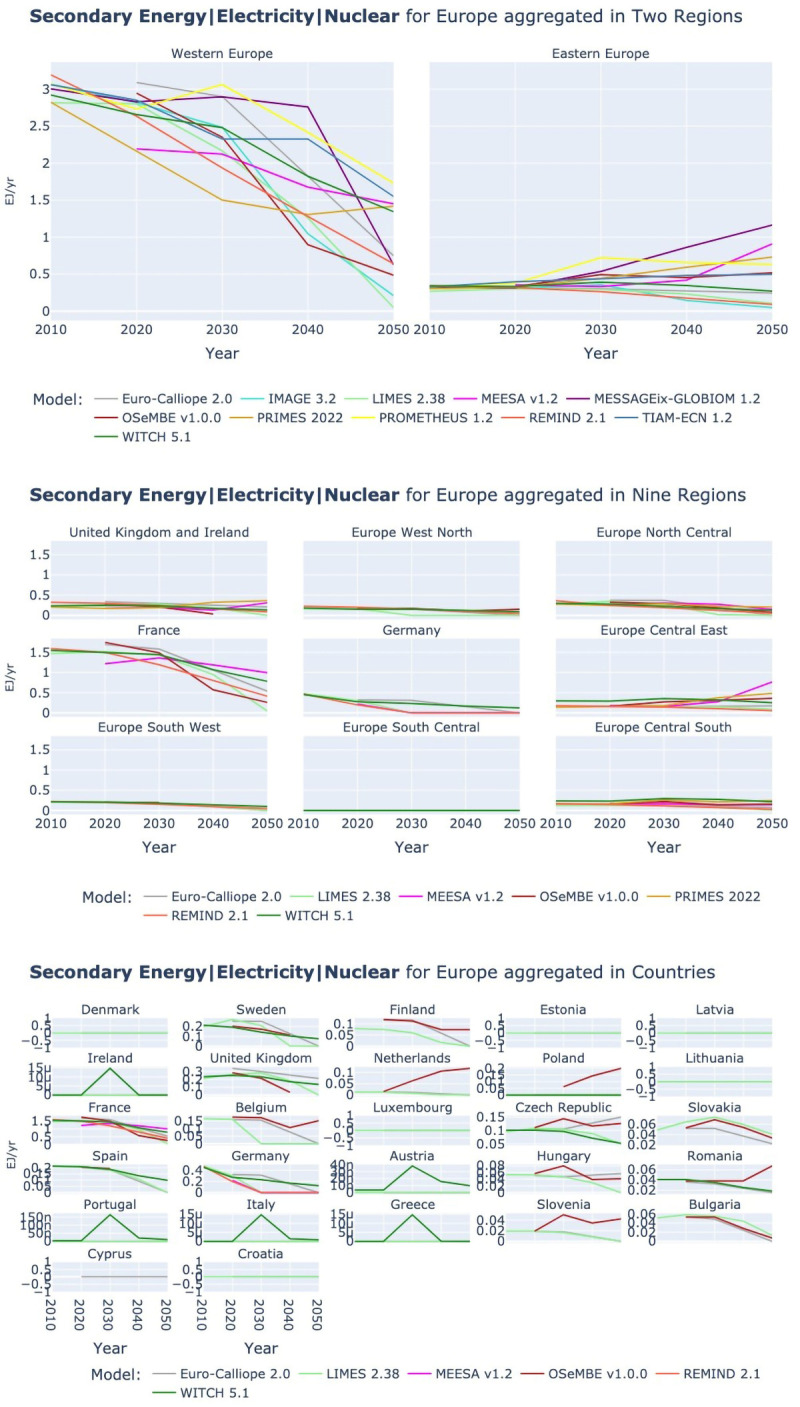
Secondary Energy Electricity Nuclear at three aggregations across models. Two region aggregation at the top, nine region aggregation in the middle, and country resolution at the bottom.

Due to the lack of carbon emissions during its operation, nuclear power has experienced a revival in some European national energy debates in recent times. However, the picture for nuclear is at the best to be described as mixed. In Eastern Europe the trends for nuclear power generation are not consistent across models. Most models expect an increase or stable production of nuclear power till 2050, but some instead show a slow decline. In contrast, in Western Europe all models expect a decline of nuclear-based power generation (see Figure 12). The plots at country-resolution show that some models seem not to consider national policies regarding nuclear phase-out. This points to the potentially different assumptions around nuclear power which aren’t compared here, in particular related to costs.

### 3.3 Hydrogen

**Figure 13.**
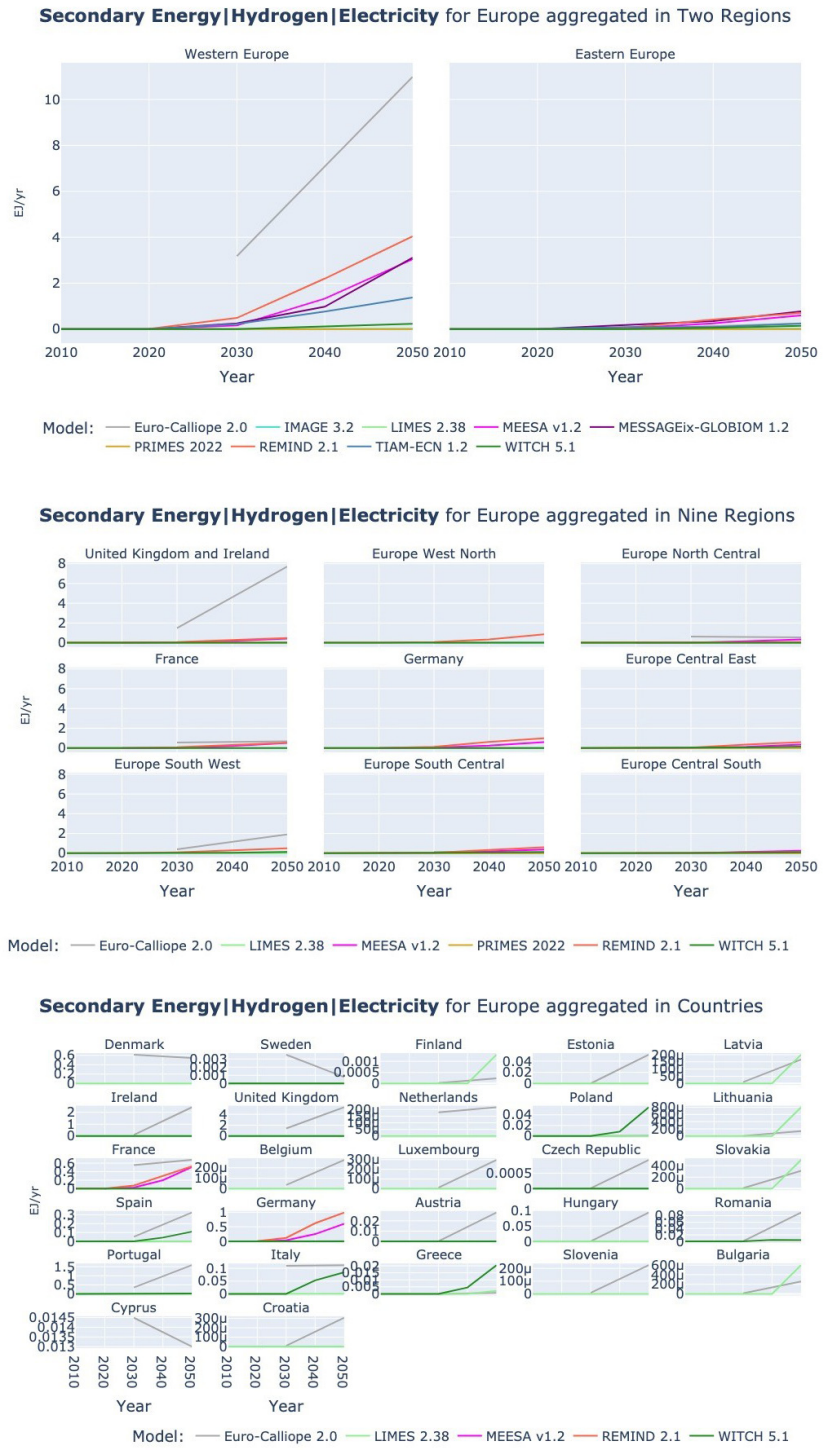
Secondary Energy Hydrogen Electricity at three resolutions across models. Two region aggregation at the top, nine region aggregation in the middle, and country resolution at the bottom.

Figure 13 shows hydrogen production by electrolysis across models and regional aggregation. In the two-region resolution we can observe that the models show a wide spread of hydrogen production levels in 2050 in Western Europe. The nine-region resolution then allows us to see that, while Euro-Calliope – the model with the highest hydrogen production – expects most of this production to happen in the UK and Ireland, the other models with high levels of hydrogen production (namely MEESA and REMIND) expect the major part of this production to happen in France and Germany, and not in renewable rich countries like Spain, Norway, or Ireland and the UK. Another interesting observation is that the WITCH model shows very low levels of hydrogen production, while we observed in the previous sections that WITCH expects a higher electricity demand – see also Figure 6.

## 4 Discussion

In the previous sections we first mapped out the different region aggregations across models, identified variables that are reported by both IAMs and ESMs and presented the results comparison for some of the jointly reported variables. The variables we presented are from three categories: demands, electricity supply, and hydrogen – an energy carrier that is expected be used in multiple ways in a decarbonised energy system.

The mapping of the different region aggregations showed that there are predominant model region aggregations with the EU aggregated into two or nine regions, but that the actual aggregation of countries often varies from model to model, highlighted in Figure 2 by the marked harmonised regions. This prevents an accurate and detailed model comparison. For example, how the difference in aggregation between PRIMES and MEESA and REMIND regarding Germany and France hampers the model comparison becomes visible in Figure 6. The nine-region resolution used by MEESA and REMIND shows very similar levels of final electricity demand across regions. Integrating Germany and France into other regions would distort this.

We also note that IAMs more commonly have a more aggregated approach than ESMs – see Figure 2. These variations are an obstacle for consistent model comparisons which could be overcome with better coordination across modelling teams to use harmonised native regions, while moving beyond the EU-wide assessments and provide more disaggregated information on key decarbonisation strategies.

The variable mapping shows for the power sector that both types of models provide results at the same level of detail by energy carrier.

For the demand side, heat, and hydrogen the mapping shows that the compared ESMs seem to be more aggregated in comparison to the involved IAMs – see Table 2. But, at least in the case of Euro-Calliope, this is not the case. Euro-Calliope actually models heat at high detail and distinguishes between different technological supply options like district heating vs. stand-alone. However, it does not distinguish between economic sectors. Therefore, it reports only one type of heat, when using the IMAC-nomenclature.

Nevertheless, for ESMs with a limited sectoral coverage, hydrogen demands derived from IAMs and full system ESMs could be a variable that could be used as an input. Another option for information flow between models could be the above-mentioned sectoral demands from IAMs and full system models. But, as we noticed for the case of heat in Euro-Calliope, the issue here might not be that the models are not modelling certain demands with more detail but rather with different detail, e.g., instead of by economic sector with higher technological resolution. In such a case a model linking might be difficult, but potentially a comparison of the different representations would bring benefits for both model types. ESMs could refine their representation knowing about economic sectors, and IAMs could refine their representation of technological detail.

In the results for Final Energy and Final Energy Electricity, we observe a rather homogenous picture without major outliers by any model, with consistent trends towards increased electrification of energy end-uses. In the second results category analysed – the energy sources for power generation – significant differences across models are observed. However, the differences are not explained by the model type, as shown they are to some extent linked to the varying levels of demand across models, but most likely also to different modelling assumptions regarding costs and resource availability, which are not compared in this paper. The same is the case for the results on the production of hydrogen via electrolysis, models differ notably in the expected amount of hydrogen produced, but the differences are not correlated with the model type. The differences in power generation and hydrogen production can partly be linked to different demand levels in the models, but partly they also indicate different constraints regarding resource availability, technology costs, and operational constraints.

## 5 Conclusions

In conclusion the work conducted for this paper highlights the following:

- Despite region aggregations with similar number of regions, IAMs and ESMs differ in the aggregation of countries to regions, which hampers direct model comparison.
- A model comparison of a wide range of variables across different regional aggregations can identify and trace differences in results between models to their origin.
- Variable mapping can facilitate the identification of commonly reported variables and can thereby ease model comparisons. It also facilitates the identification of possible information flows between models of different sectoral coverage.

Common standards for region aggregation could facilitate model comparison exercises. Identifying harmonised regions through a mapping exercise, as conducted for this paper, can help lead to a more effective comparison of results. We highlight two levels of region aggregation across which ESMs and IAMs can be compared. The two-region level is the most aggregate and allows the comparison of all models in the comparison. But removes some of the detailed insights from the ESMs. The nine-region level provides a greater opportunity for comparison with ESMs, because it allows a better consideration of regional differences in resource availability and demand, while reducing the computational effort that comes along with a country resolution. However, the varying region aggregations highlighted by the attempt to define harmonised regions in Figure 2 represent an obstacle for detailed model comparisons. A potential approach in future to define harmonised regions could involve optimisation techniques. This would allow to systematically consider different dimensions of the decision on how to group countries to regions.

The mapping of reported variables is a simple analysis of the data reported by models in a model comparison. But simple as it might be, it facilitates the usage of the reported data for analysis and facilitates the later addition of other models to the comparison by giving an overview of what the most common variables reported are. Therefore, a conclusion of this paper is that platforms such as the IIASA Scenario Explorer – that has been used for the work presented here – could increase the likelihood that their database will be further used and expanded after initial project funding has ended by providing statistics on how many models have reported a variable. This allows modelling teams that are adding their results later and that are perhaps not even part of the initial project to better identify what are the core variables to report. Policy makers would also obtain a better understanding of what insights models do deliver and how well that aligns with what they consider relevant.

The variable comparison highlights that the sectoral coverage of the compared IAMs and ESMs differs, but also that there is an overlap in reported variables. It also highlights that the IAMC-nomenclature could be expanded to allow a better consideration of the differences in modelling techniques between IAMs and ESMs, which in turn would allow more in-depth comparison.

The presented region mapping of models for the EU and UK is a novel addition to the literature by providing insights in how models define regions differently.

The comparison of IAMs commonly focuses on the EU or even global level. The here presented disaggregation provides more detailed modelling results for a decarbonisation scenario for regions within the EU. The region mapping and variable mapping together highlight that for standardised model comparisons and potential model linking a better harmonisation of region aggregations and information on commonly reported variables and their meaning is required. This underlines the relevance of the ECEMF project and its objective of providing an open-source full scale model comparison to the European modelling community.

## Data Availability

The data underlying this study is available at: https://data.ece.iiasa.ac.at/ecemf Zenodo: ECEMF Diagnostic Scenarios, version 1.0. https://doi.org/10.5281/zenodo.7634845 ( Henke, 2023a) This project contains the following underlying data: ECEMF-diagnostic-scenarios-v1.xlsx (Archived underlying data at time of publication. Zenodo: Interactive plots for all variables identified in Table 2 that are reported by more than two models, at three resolutions. https://doi.org/10.5281/zenodo.7640847 ( Henke, 2023b). Data are available under the terms of the Creative Commons Attribution 4.0 International license (CC-BY 4.0). Repository: https://github.com/HauHe/ESMsxIAMs_figs/tree/v0.1.1 Webpage: https://hauhe.github.io/ESMsxIAMs_figs/ List of variables with available plots: **Emissions** • CO2|Energy|Supply|Electricity • CH4|Energy|Supply **Demands** (Final Energy) • Final Energy • Electricity • Residential and Commercial • Residential and Commercial|Electricity • Commercial • Commercial|Electricity • Residential • Residential|Electricity • Transportation • Transportation|Electricity **Primary Energy** • Biomass|Electricity • Coal|Electricity • Gas|Electricity • Oil|Electricity **Electricity Supply** (Secondary Energy|Electricity) • Biomass • Coal • Gas • Geothermal • Hydro • Nuclear • Ocean • Oil • Solar • Solar|CSP • Solar|PV • Wind • Wind|Offshore • Wind|Onshore **Heat** • Final Energy|Heat • Secondary Energy|Heat **Hydrogen** • Final Energy|Hydrogen • Secondary Energy|Hydrogen • Secondary Energy|Hydrogen|Electricity

## Software availability

Source code available from: https://github.com/HauHe/ESMsxIAMs/tree/v0.3.2

Archived source code at time of publication: https://doi.org/10.5281/zenodo.7640846 ( Henke, 2023a)

License: MIT
