# Comparing energy system optimization models and integrated assessment models: Relevance for energy policy advice

**DOI:** 10.12688/openreseurope.15590.2

**Published:** 2024-03-21

**Authors:** Hauke Henke, Mark Dekker, Francesco Lombardi, Robert Pietzcker, Panagiotis Fragkos, Behnam Zakeri, Renato Rodrigues, Joanna Sitarz, Johannes Emmerling, Amir Fattahi, Francesco Dalla Longa, Igor Tatarewicz, Theofano Fotiou, Michał Lewarski, Daniel Huppmann, Kostas Kavvadias, Bob van der Zwaan, Will Usher

**Affiliations:** 1 Division of Energy Systems, KTH Royal Institute of Technology, Stockholm, 10044, Sweden; 2 Copernicus Institute of Sustainable Development, Utrecht Universiteit, Utrecht, The Netherlands; 3 Planbureau voor de Leefomgeving, Den Haag, The Netherlands; 4 Faculty of Technology, Policy and Management, Delft University of Technology, Delft, The Netherlands; 5 Potsdam Institute for Climate Impact Research, Potsdam, Germany; 6 E3-Modelling S.A., Panormou 70-72, Athens, Greece; 7 Energy, Climate, and Environment (ECE) Program, International Institute for Applied Systems Analysis (IIASA), Laxenburg, Austria; 8 Global Energy Systems Analysis, Technische Universität Berlin, Berlin, Germany; 9 RFF-CMCC European Institute for the Economics and the Environment (EIEE), Milan, Italy; 10 TNO Energy Transition, Amsterdam, The Netherlands; 11 The Institute of Environmental Protection – National Research Institute (IOS-PIB) / National Centre for Emissions Management (KOBiZE), Warsaw, Poland

**Keywords:** Net-zero scenario analysis, Climate change mitigation, Renewable energy transition, power system, open-source

## Abstract

**Background:**

The transition to a climate neutral society such as that envisaged in the European Union Green Deal requires careful and comprehensive planning. Integrated assessment models (IAMs) and energy system optimisation models (ESOMs) are both commonly used for policy advice and in the process of policy design. In Europe, a vast landscape of these models has emerged and both kinds of models have been part of numerous model comparison and model linking exercises. However, IAMs and ESOMs have rarely been compared or linked with one another.

**Methods:**

This study conducts an explorative comparison and identifies possible flows of information between 11 of the integrated assessment and energy system models in the European Climate and Energy Modelling Forum. The study identifies and compares regional aggregations and commonly reported variables. We define harmonised regions and a subset of shared result variables that enable the comparison of scenario results across the models.

**Results:**

The results highlight how power generation and demand development are related and driven by regional and sectoral drivers. They also show that demand developments like for hydrogen can be linked with power generation potentials such as onshore wind power. Lastly, the results show that the role of nuclear power is related to the availability of wind resources.

**Conclusions:**

This comparison and analysis of modelling results across model type boundaries provides modellers and policymakers with a better understanding of how to interpret both IAM and ESOM results. It also highlights the need for community standards for region definitions and information about reported variables to facilitate future comparisons of this kind. The comparison shows that regional aggregations might conceal differences within regions that are potentially of interest for national policy makers thereby indicating a need for national-level analysis.

## 1 Introduction

Models of all kinds, scopes and goals are increasingly used in energy and climate policy advice and systems design at all scales, from global and regional down to national and sub-national scale. For example, integrated assessment models (IAMs) provide insights on the interactions between energy systems, the economy, land-use, and climate, increasingly needed for informing long-term policy making. Energy system optimisation models (ESOMs), instead, provide in-depth and context-specific insights on the technological transition required to decarbonize the energy system with commonly more detailed representation of temporal, spatial, technological, and operational aspects. For each of the two types of models, a body of literature has been built that compares and links complementary models. The comparison of models and their results commonly serves the purpose of better understanding the differences in the results of models, or of providing additional insights. The differences can be structural, i.e., how do model and modelling framework represent the world, or parametric, which means what state of the world does the model represent based on the input data, the selected value of model parameters, and hence the boundary conditions. Understanding the differences between models improves the understanding of whether the insights derived from the models are robust or not. At the same time, comparisons allow identifying possible synergies and opportunities for model linking, in such a way for models to complement each other’s insights and provide enhanced information.

In the field of climate modelling, systematic model comparisons and the use of comparison metrics have already had a significant history.

The fields of integrated assessment modelling and energy systems modelling aim, in contrast to climate models, to represent socio-technical and socio-economic systems and are therefore not purely relying on the laws of physics which makes reliability and validation significantly more difficult. But despite the different nature of models there are potentially lessons to be learned from the experiences of the climate modelling community. In any case, in these two fields many comparisons have also been conducted among models of the same type, e.g., among IAMs ( Harmsen *et al.*, 2021) or ESOMs ( Ruhnau *et al.*, 2022) separately. In some of these comparisons, metrics have been developed that allow a standardized comparison of models and results. Standardized comparison methods, in turn, allow repeatability and the expandability of comparison exercises, while also favouring the identification of common variables and indicators for model linking where models display the potential to provide complementary insights.

For IAMs, work has been conducted in recent years to systematically compare results across models using diagnostic indicators and diagnostic scenarios to verify the robustness of provided insights and to improve the understanding of differences in their results ( Dekker *et al.*, 2023; Harmsen *et al.*, 2021; Kriegler *et al.*, 2015). Like climate models and IAMs, ESOMs are also commonly used to inform policy processes, particularly in decarbonisation efforts. Also for ESOMs, model comparisons are a common practice, used to understand the differences in model results and derive robust insights across models which can be used for policy recommendations ( Capros *et al.*, 2014).

In contrast to the comparison of models, the linking of models connects two or more models with complementary capabilities. This can be done via a soft-link that keeps the models as independent systems that exchange variables and run iteratively until their solutions converge, or via a hard link that establishes a procedure that allows to run the models together. In the field of energy systems modelling a common link is between models that focus on capacity expansion and investment planning and models that focus on the operation of the modelled system. Linking such models increases the robustness of the results of the investment planning models ( Deane *et al.*, 2012). However, the linking can also involve a multitude of models with very different scopes. The H2020 project OpenENTRANCE developed an open modelling platform consisting of models and datasets that allow model linking and the investigation of the role of human behaviour in decarbonisation scenarios. The SENTINEL project, also a H2020 initiative, developed a platform that provides the possibility to select and link models, which when linked are suitable to answer specific questions related to decarbonisation.

Gardumi *et al.*, developed an integrated assessment framework by linking ten models of different kinds, with a pan-European ESOM and a global computable general equilibrium model at the core, linking to models covering local to national aspects of society, environment, and the energy system. The developed framework provides insights beyond the energy system for ecosystems and society across multiple geographic scales, but does not involve any IAMs ( Gardumi *et al.*, 2022).

We can note that model comparisons are commonly within a group of the same model type, while model linking is often connecting models of different type. There have been some cross-comparisons of model results across different model types, including IAMs and ESOMs ( Roelfsema *et al.*, 2020), but further formalized efforts are needed. To provide policymakers with more consistent messages, model comparisons among models of different types can contribute to a better understanding of the differences in results between these models, and the enhanced robustness of model-driven insights.

The model types that are compared in this paper, namely IAMs and ESOMs, both apply quantitative methods to model the analysed systems. In the comparison we include the six IAMs:

- Integrated Model to Assess the Global Environment (IMAGE)
- MESSAGEix-GLOBIOM
- PROMETHEUS
- Regional model of investments and development (REMIND)
- TIAM-ECN
- World induced technical change hybrid model (WITCH)

And the five ESOMs:

- Euro-Calliope
- Long-term investment model for the electricity sector (LIMES)
- The model for European energy system analysis (MEESA)
- The open source electricity model base for Europe (OSeMBE)
- The price-induced market equilibrium system (PRIMES)

IAMs model human-earth systems to generate insights into global environmental change and issues of sustainable development. Moreno *et al.* (2023) and Riahi *et al.* (2017) illustrate how IAMs are used to assess global pathways for the development of integrated resource systems and related greenhouse gas emissions. The latter developed the Shared Socioeconomic Pathways (SSPs) Riahi *et al.* (2017), also using the models IMAGE, MESSAGE, REMIND, and WITCH. The TIAM-ECN model has been used to analyse the economic, societal and energy system implications of a hydrogen partnership between Europe and North Africa ( van der Zwaan *et al.*, 2021), while PROMETHEUS has been used to assess the energy and emission impacts of NDCs and long-term Paris Agreement goals ( Fragkos & Kouvaritakis, 2018).

In contrast to IAMs, ESOMs represent the energy system or sub-sectors of it, investigating the long-term technology deployment options and investment cycles or detailed system operation with the representation of individual countries or sub-national regions and temporal resolution of years to hours ( Pfenninger *et al.*, 2018). Tröndle (2020), for example, uses the ESOM Euro-Calliope to investigate the trade-offs between using renewable energies locally or at the sites of the best resources and Pickering *et al.* (2022) use Euro-Calliope to identify near-optimal solutions for a decarbonised European energy system. The LIMES model, developed at the Potsdam Institute for Climate Impact Research (PIK), has been used to investigate the effect of the new EU Green Deal targets on the EU Emission Trading System ( Pietzcker *et al.*, 2021) and the interactions with the Market Stability Reserve ( Osorio *et al.*, 2021). Of the group of compared models, the two ESOMs OSeMBE and MEESA have so far been least applied in the literature. OSeMBE is built using the open-source modelling framework OSeMOSYS ( Henke *et al.*, 2022). The MEESA model is based on OSeMOSYS as well, but uses a translation of the source code to GAMS and a modified set of equations ( Tatarewicz *et al.*, 2022).

In summary, the two model types differ in their scope and resolution, with IAMs providing global insights across a substantial proportion of the economy, but at a higher regional aggregation and a cruder temporal and technology resolution. However, the IAM PROMETHEUS and the ESOM PRIMES have been repeatedly used to provide energy reference scenarios for the European Commission and thereby highlight that these two model types can complement each other.

The aim of this study is to describe the overlaps between integrated assessment and energy system models in the context of modelling possible European decarbonisation pathways and how these overlaps might vary depending on the model implementation. Comparing IAMs and ESOMs at the same time, has the potential to bring about novel and urgently needed insights. For instance, in terms of the compatibility of long-term energy policies with the technical requirements of the energy system operation. Such comparisons across model types have been rarely realised, leading to a lack of agreement in terms of the viability of alternative energy transition strategies. Therefore, we want to focus here on the simultaneous comparison between IAMs and ESOMs.

To achieve the aim of this study we follow three research questions. The first research question (RQ1) of methodological nature is if harmonized regions can be defined, and if there are commonly reported variables? We attempt to answer this research question in the next section identifying harmonised region aggregations and result parameters which allow the comparison of the IAMs and ESOMs from the European Climate and Energy Modelling Forum (ECEMF) project included in this study. The second research question (RQ2) is what are the differences in the results of the compared models? And can they be related to structural reasons or parametric cause? With the mapping of regions and variables from the first research question, we respond to the second research question by analysing the results of a diagnostic deep-decarbonisation scenario run by all the models. The third research question (RQ3) is how could the two assessed model types benefit from each other? To answer RQ3 we investigate the possibilities to exchange information across models, i.e., for what aspects can the models inform each other, for instance, with regards to different sectoral demands not covered by (some) ESOMs, and rates of technology implementation over time and space.

## 2 Methodology

**Figure 1.**
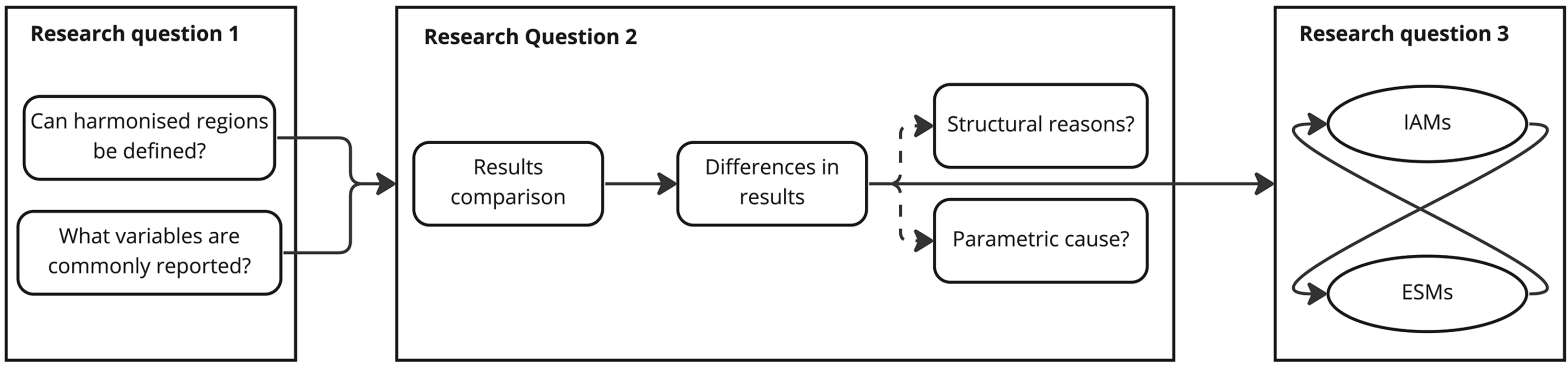
Linking research questions to methods.

In this section, we describe in detail the steps taken to meet the aim of the paper. In Section 2.1, we outline the selection criteria for including models in the study and describe the design of the diagnostic scenario used in this study. In Section 2.2, we describe the process by which we arrived at three levels of harmonised regions we can use to compare the model results. In Section 2.3 we describe the procedure used to identify common reporting variables. Figure 1 illustrates how the research questions are structured into sub-questions and steps, and how the sub-questions build up on each other to answer the overarching questions.

### 2.1 Selection of models for comparison

In the model comparison eleven IAMs and ESOMs are compared. Table 1 provides an overview of the models, the compared version, the type, the regional aggregation of EU and UK, and a reference to their documentation.

**Table 1.** Integrated Assessment Models (IAMs) and Energy System Optimisation Models (ESOMs) in comparison exercise. Detailed descriptions of the IAMs and the option to compare their model design and logic are available at https://www.iamcdocumentation.eu/index.php/Model_comparison.

Abbr.	Model	Version	Type	Europe resolution	Documentation/website
EUR	Sector-Coupled Euro-Calliope	1.0	ESOM	Country	( Pickering *et al.*, 2022)
IMA	IMAGE	3.2	IAM	2-region	https://models.pbl.nl/image/index.php/Welcome_to_IMAGE_3.2_Documentation
LIM	LIMES	2.38	ESOM	Country	https://www.pik-potsdam.de/en/institute/departments/transformation-pathways/models/limes
MEE	MEESA	1.1	ESOM	9-region	( Tatarewicz *et al.*, 2022)
MES	MESSAGEix- GLOBIOM	1.2	IAM	2-region	https://docs.messageix.org/projects/global/en/latest/
OSE	OSeMBE	1.0	ESOM	Country	https://osembe.readthedocs.io/en/latest/
PRI	PRIMES	2022	ESOM	7-region	https://e3modelling.com/modelling-tools/primes/
PRO	PROMETHEUS	1.2	IAM	2-region	https://e3modelling.com/modelling-tools/prometheus/
REM	REMIND	3.0.0	IAM	9-region	( Baumstark *et al.*, 2021)
TIA	TIAM-ECN	1.2	IAM	2-region	https://www.iamcdocumentation.eu/index.php/TIAM-ECN
WIT	WITCH	5.1	IAM	18-region	https://www.witchmodel.org/

In the ECEMF project a set of diagnostic scenarios has been developed ( Dekker *et al.*, 2023). These scenarios aim to bring models into extreme states to explore their behaviour. However, in this paper the goal is to explore the overlaps and potential for linking IAMs and ESOMs. To do so we believe it is sufficient to analyse the results of one scenario. We select a diagnostic scenario with a high carbon price called DIAG-C400-lin with the carbon price increasing to 400 US$ in 2040 and to 580 US$ in 2050. This implies that we are analysing a scenario with deep decarbonisation towards 2050, i.e., the direction EU policy makers are aiming for. In the global models the assumptions for GDP and population development have been harmonized matching the SSP2 scenarios to ensure a broad consistency across models ( ECEMF, 2022). Furthermore, for historic years a comparison of model results and numbers reported by Eurostat has been conducted and models have been aligned to the historic developments as far as possible.

### 2.2 Mapping model regions to harmonised regions for comparison

For both model types, it is widespread practice to define native model regions. These native regions aggregate collections of countries for which results are reported by the models. Models use aggregation to reduce computational demands. Some models, such as PRIMES, are specified at more detailed regional aggregation than that which their results are available. Especially among ESOMs, some models report at more detailed spatial granularity, e.g., at the country or sub-country scale in the EU. The aggregation of countries to native regions can happen with different objectives in mind which as we show later creates differences across models, even when presenting the same European resolution. However, model results can only be compared when harmonised regions are identified. We define the following rules to identify a model region:

- A model must define one or more regions consisting of one or more countries.
- A country can only appear in one region.

Harmonised regions are defined as regions that appear in two or more models that contain the same countries. It is important to note that two models may use the same name for their model regions, but the pattern of countries contained do not match. It was necessary in this study to relax the strict definition of “exact match” to “or with a significant number of the same countries”. The definition of harmonised regions is not an explicitly spatial approach, but a way to define common aggregations for model nodes that represent regions of countries or individual countries.

**Figure 2.**
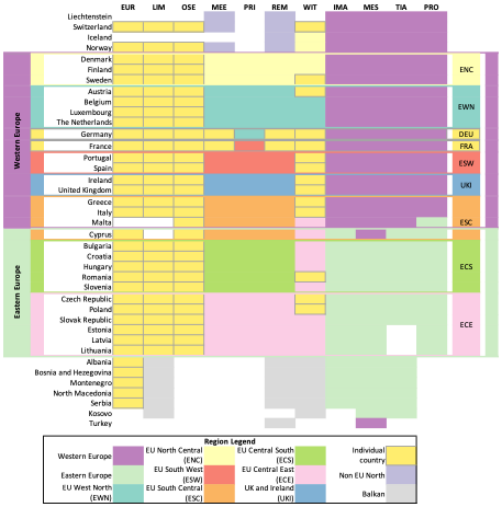
Region mapping for the EU27 & UK. The abbreviations used instead of model names are listed in Table 1.

**Figure 3.**
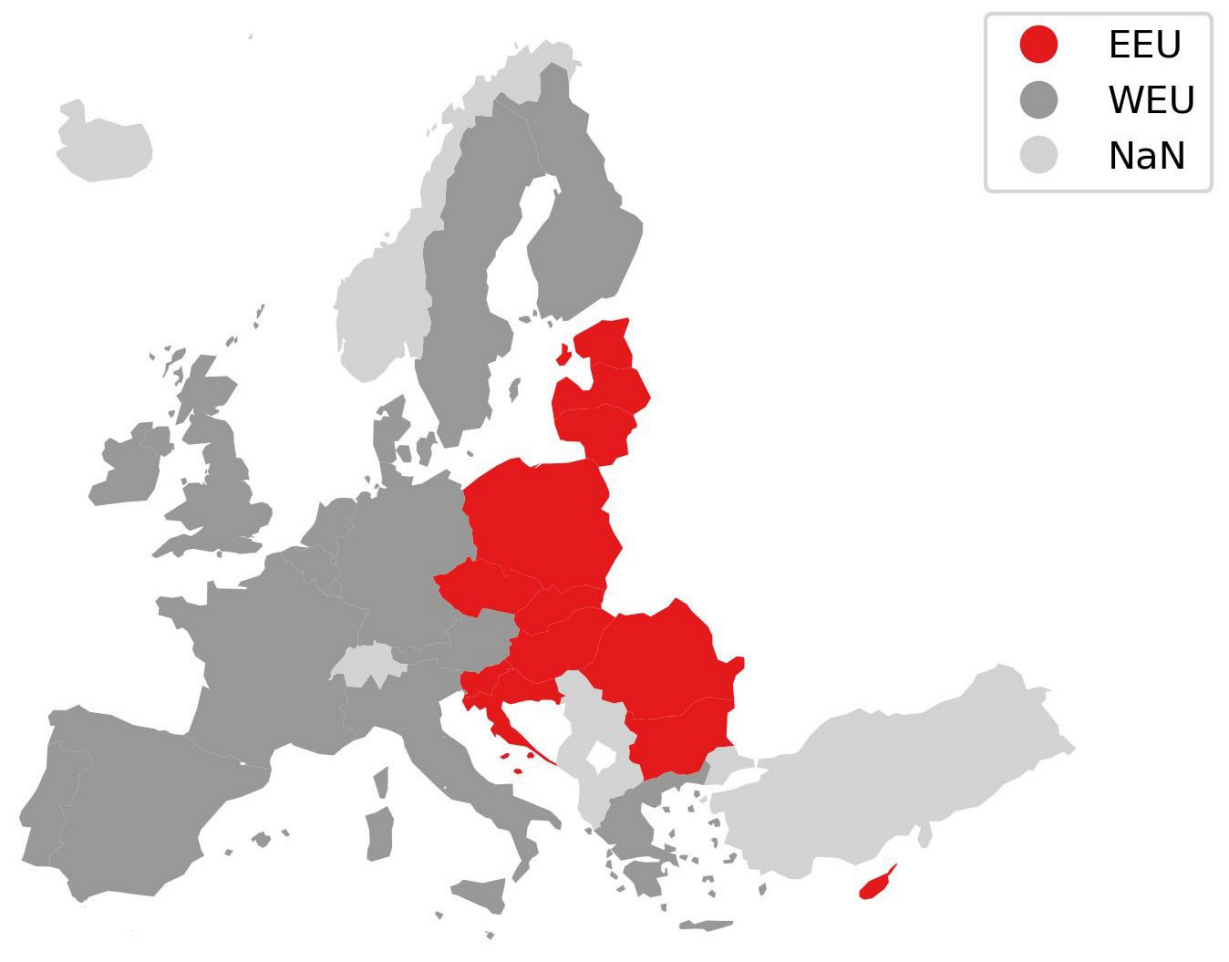
Harmonised two region mapping.

**Figure 4.**
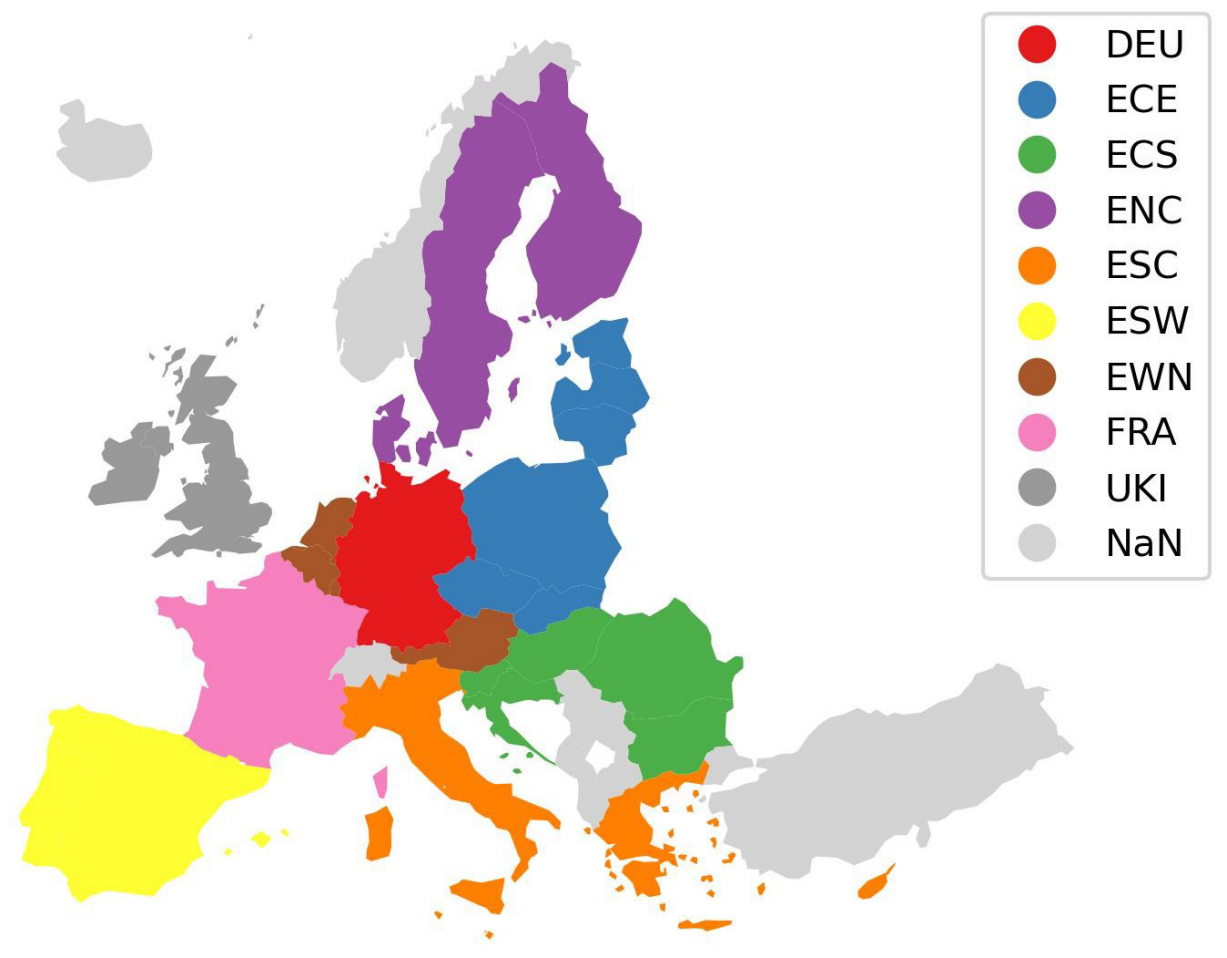
Harmonised nine region mapping.

To define harmonised regions for this paper, the first step was to collect the information on how the models involved in the comparison aggregate countries to native regions from model documentations (see Table 1 for references to documentation) and model mapping in the openENTRANCE Python package. In the second step the identified region aggregations are compared across models in tabular form and by visualising the region mapping. Lastly, based on this comparison harmonised regions are defined, that allow the comparison of as many models as possible at distinct levels of aggregation. The results of this process are documented comprehensively in Figure 2. The harmonised regions are additionally also shown in Figure 3 and Figure 4.

Figure 2 lists the models in the columns, and the countries in the rows. On the very left and right the derived harmonised regions are marked. Western and Eastern Europe are marked in purple and mint green. The horizontal lines between Norway and Denmark, Malta and Cyprus, and Lithuania and Albania mark the boarders of the harmonised regions of Western and Eastern Europe. The filled cells in the four columns on the right side of the figure for IMAGE, MESSAGEix-GLOBIOM, PROMETHEUS, and TIAM-ECN indicate which countries they consider part of Western and Eastern Europe. The two regions are to a large extent identical across the four models. The most significant difference is that MESSAGE-WEU includes Turkey, while the European regions of IMAGE, PROMETHEUS, and TIAM-ECN do not. Furthermore, the models vary in allocating the island countries Malta and Cyprus, which however are small and have a limited contribution in EU-wide pathways.

The second kind of aggregation is visible when looking at MEESA, PRIMES, and REMIND in the central columns of the figure. The most significant difference here is that MEESA and REMIND represent France and Germany as one-country-region each, while PRIMES integrates France and Germany respectively into the regions EU South West (ESW) and EU West North (EWN). MEESA and REMIND aggregate the remaining countries of the EU and UK into seven regions of two to six countries. This country aggregation was first implemented in the REMIND model by Rodrigues *et al.* (2022). Beyond the EU and UK, MEESA and REMIND also model and report results for “Non-EU North,” the Balkan countries, and Turkey; since they are outside the EU and UK region, they are not considered in this model comparison.

Thirdly, the mapping exercise in Figure 2 illustrates the approach taken by the WITCH model. In WITCH, 13 countries within the EU27 and the UK are modelled as single country regions (marked in yellow with a grey frame in Figure 2), while the remaining fifteen countries are aggregated into three regions with EU member states. Furthermore, Switzerland is modelled as a single country and the Balkan countries as one region.

Lastly, on the left side in Figure 2 are the models Euro-Calliope, LIMES, and OSeMBE. These three models do not aggregate the modelled countries to regions, but model each of the European countries individually or even at sub-national level in the case of Euro-Calliope (not shown in Figure 2).

In summary, the region mapping illustrates that there are four kind of resolutions that are used to model the EU and UK in the IAMs and ESOMs involved in this comparison, namely aggregating countries into two regions (commonly Western and Eastern EU), grouping them into nine regions, grouping smaller countries into four regions and modelling the rest individually, and modelling countries individually. However, it is notable that models tend to vary in the allocation of countries. This can limit the comparability of results across models.

For the comparison in this paper, we derive two harmonised region aggregations which are marked in Figure 2. Figure 3 shows the harmonised two region aggregation for the EU and UK used in this paper. Cyprus is considered part of Eastern Europe and Malta is considered part of Western Europe, both cases follow the majority of “two-region” models compared. Turkey is not considered for the comparison.

Figure 4 shows the second harmonised region aggregation derived based on the aggregations used by the models MEESA, PRIMES, and REMIND aggregating the EU and the UK into nine regions. In the harmonised region aggregations the following regions exist:

- Harmonised two-region aggregation
  - Western Europe
  - Eastern Europe
- Harmonised nine-region aggregation
  - EU West North (EWN)
  - EU North Central (ENC)
  - EU South West (ESW)
  - EU South Central (ESC)
  - EU Central South (ECS)
  - EU Central East (ECE)
  - Germany (DEU)
  - France (FRA)
  - UK and Ireland (UKI)

An alternative approach to the manual mapping conducted here would be to use an explicitly spatial approach, mapping native regions to polygons representing the areas covered. Where differences, such as an overlap, are identified in aggregate regions between models, a spatial join or interpolation based on proxy variables (such as GDP and population for final energy demand) could be used to extract results for the individual country. However, this would be a much more labour-intensive approach and introduces considerable uncertainty and methodological complexity into the process of comparing results. In this first of its kind analysis, we limited the comparison to the presented harmonised regions.

### 2.3 Identifying common reporting variables

In parallel to identifying harmonised regions, we embarked on an investigation of common variables across both ESOMs and IAMs. The reporting standard in the ECEMF project follows the IAMC-format, defined in the community-wide used database managed by IIASA and extensively used in many model intercomparison projects and in IPCC AR6 ( Huppmann *et al.*, 2021). In total, there are over 1,000 variables defined in the IAMC template, but only a subset of these are relevant to this study. The variables in IAMC-format can be both model inputs or outputs depending upon the model, and a variable that is an output for one model can be an input for another model.

The process of identifying common variables is manual, and was performed by examining the uploaded scenario data provided for the diagnostic scenario. However, modelling teams are continually updating their reporting of variables, and may add or remove variables over time. As such, the variables reported here are not necessarily representative of all the outputs available from the included models ( Cherp *et al.*, 2021).

Table 2 shows a list of identified common IAMC-format variables that are reported by IAMs and ESOMs in the ECEMF project. They are selected to explain the most important aspects of the full energy (supply and demand) system.

**Table 2.** Variable mapping. The abbreviations for the model names are listed in Table 1.

	ESOMs					IAMs					
Variable	EUR	LIM	MEE	OSE	PRI	IMA	MES	REM	PRO	TIA	WIT
**Capacity**											
Electricity|**	No	Yes	No	Yes	No	No	No	No	No	No	No
**Emissions**											
CO2|Energy|Supply|Electricity	No	Yes	Yes	Yes	Yes	Yes	Yes	Yes	Yes	Yes	Yes
CH4|Energy|Supply	No	No	No	No	Yes	Yes	No	Yes	No	Yes	Yes
Kyoto Gases|Energy	No	No	No	No	Yes	No	No	Yes	No	No	No
**Demands (Final Energy)**											
Final Energy	Yes	No	No	No	Yes	Yes	Yes	Yes	Yes	Yes	Yes
Electricity	Yes	No	No	Yes	Yes	Yes	Yes	Yes	Yes	Yes	Yes
Residential and Commercial	No	No	No	No	No	Yes	Yes	Yes	Yes	Yes	Yes
Residential and Commercial|Electricity	No	No	No	No	No	Yes	Yes	Yes	Yes	Yes	Yes
Commercial	No	No	No	No	Yes	Yes	No	No	No	Yes	Yes
Commercial|Electricity	No	No	No	No	No	Yes	No	No	No	Yes	Yes
Residential	No	No	No	No	No	Yes	No	No	No	Yes	Yes
Residential|Electricity	No	No	No	No	No	Yes	No	No	No	Yes	Yes
Transportation	Yes	No	No	No	Yes	Yes	Yes	Yes	Yes	Yes	Yes
Transportation|Electricity	Yes	No	No	No	Yes	Yes	Yes	Yes	Yes	Yes	Yes
**Primary Energy**											
Biomass|Electricity	No	Yes	Yes	Yes	Yes	Yes	No	Yes	No	No	No
Coal|Electricity	No	Yes	Yes	Yes	Yes	Yes	No	Yes	No	No	No
Gas|Electricity	No	Yes	Yes	Yes	Yes	Yes	No	Yes	No	No	No
Oil|Electricity	No	Yes	Yes	No	Yes	Yes	No	No	No	No	Yes
**Electricity Supply (Secondary** ** Energy|Electricity)**									
Biomass	Yes	Yes	Yes	Yes	Yes	No	Yes	Yes	Yes	Yes	Yes
Coal	Yes	Yes	Yes	Yes	Yes	Yes	Yes	Yes	Yes	Yes	Yes
Gas	Yes	Yes	Yes	Yes	Yes	Yes	Yes	Yes	Yes	Yes	Yes
Geothermal	No	No	No	Yes	Yes	Yes	Yes	Yes	No	Yes	No
Hydro	Yes	Yes	Yes	Yes	Yes	Yes	Yes	Yes	Yes	Yes	Yes
Nuclear	Yes	Yes	Yes	Yes	Yes	Yes	Yes	Yes	Yes	Yes	Yes
Ocean	No	No	No	Yes	Yes	No	No	No	No	No	No
Oil	No	Yes	Yes	Yes	Yes	Yes	Yes	Yes	Yes	Yes	Yes
Solar	Yes	Yes	Yes	Yes	Yes	Yes	Yes	Yes	Yes	Yes	Yes
Solar|CSP	No	Yes	No	No	Yes	Yes	Yes	Yes	No	Yes	Yes
Solar|PV	Yes	Yes	Yes	Yes	Yes	Yes	Yes	Yes	No	Yes	Yes
Wind	Yes	Yes	Yes	Yes	Yes	Yes	Yes	Yes	Yes	Yes	Yes
Wind|Offshore	Yes	Yes	Yes	Yes	Yes	Yes	Yes	Yes	Yes	Yes	Yes
Wind|Onshore	Yes	Yes	Yes	Yes	Yes	Yes	Yes	Yes	Yes	Yes	Yes
**Heat**											
Final Energy|Heat	No	No	No	No	Yes	Yes	Yes	Yes	Yes	Yes	No
Secondary Energy|Heat	Yes	No	No	No	Yes	Yes	Yes	Yes	No	Yes	No
**Hydrogen**											
Final Energy|Hydrogen	No	No	No	No	Yes	Yes	Yes	Yes	Yes	Yes	Yes
Secondary Energy|Hydrogen	Yes	No	No	No	Yes	Yes	Yes	Yes	No	Yes	Yes
Secondary Energy|Hydrogen|Electricity	Yes	Yes	Yes	No	Yes	Yes	Yes	Yes	No	Yes	Yes

The variable mapping determined that the power sector is the main set of IAMC variables that are shared by both IAMs and ESOMs. Table 2 shows that there are few variables in the mapping that are not related to the power sector. The variables in the mapping can be grouped into seven categories: capacity, emissions, final energy demands, primary energy, electricity supply, heat, and hydrogen.

Another insight Table 2 provides is the lower detail that ESOMs provide for demand side variables. With the exception of PRIMES, none of the other ESOMs report Final Energy for the residential and or commercial sector or Heat, and also final energy in transport is only reported by Euro-Calliope and PRIMES.

It is also notable that most ESOMs do not fully report hydrogen and heat related variables. Even though, all ESOMs apart from LIMES and OSeMBE cover heat at least partly and only OSeMBE does not model hydrogen. This is a challenge when investigating the synergies offered by sector coupling, the likely essential role of hydrogen ( van der Zwaan *et al.*, forthcoming), and possible scenarios arising from electrification, e.g., increasing use of heat pumps for heat generation.

## 3 Results

The reported variables are compared across models for the harmonised regions. At the lowest regional aggregation, only those models at country scale are compared. At medium aggregation, results for lower aggregations are either summed (e.g. emissions) or averaged (prices), and models whose native regions match the harmonised regions are included. Where a mismatch occurs, the results are excluded for the harmonised region. At the highest aggregation, i.e., the two-region aggregation, all models are included in the comparison. We created plots for each of the three harmonized region aggregations for the 11 models, for each of the common reporting variables.

In this section we present results for four central aspects of decarbonisation scenarios. These aspects are ‘power generation’, ‘hydrogen production’, the ‘role of variable renewables’ and the ‘role of nuclear power’.

The plots include an indication of whether the variable values sit within a high, medium, or low range in 2050. The medium range is defined as the range from plus one standard deviation from the median to minus one standard deviation from the median. We consider the values in 2050 as high if they are higher than the median plus one standard deviation. And we consider values lower than median minus one standard deviation as low. In the plots these ranges are illustrated in a bar in 2050, where the high range is indicated in red, the medium range in yellow, and the low range in blue.

### 3.1 Overall power generation

The analysis starts from the power generation, indicated by the variable ‘Secondary Energy|Electricity’. All models in the comparison cover this variable.

**Figure 5.**
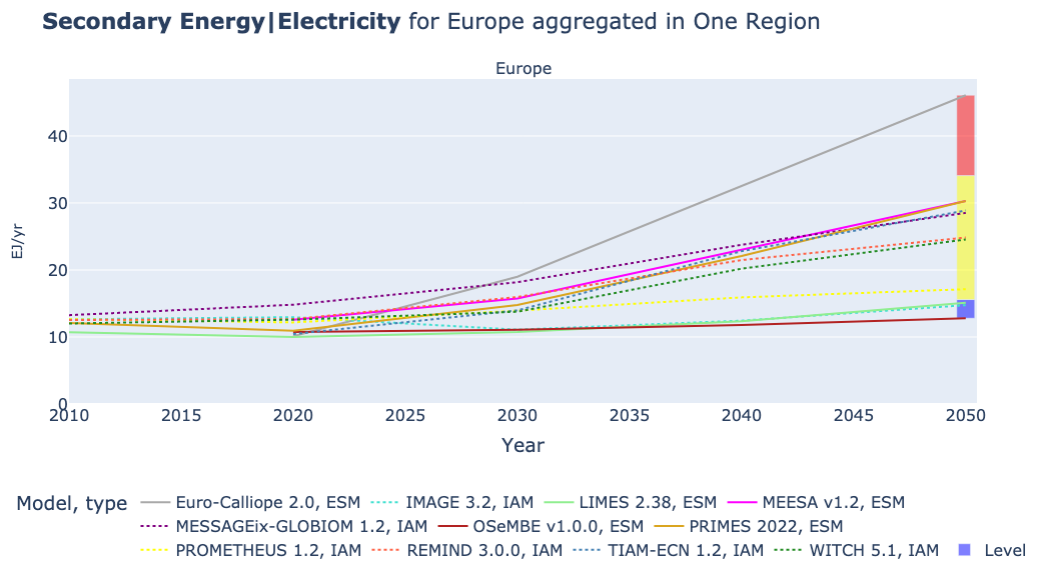
Electricity generation in Europe in one region.

In Figure 5 we can observe a wide range for the expected power generation in Europe in 2050. The figure shows six models in a medium range between 19 and 38 EJ per year. Four models are in the low range between 13 and 18 EJ per year, while Euro-Calliope sets the maximum value of 46 EJ of power generation per year. However, this level of aggregation does not provide insights on the origins of the differences.

**Figure 6.**
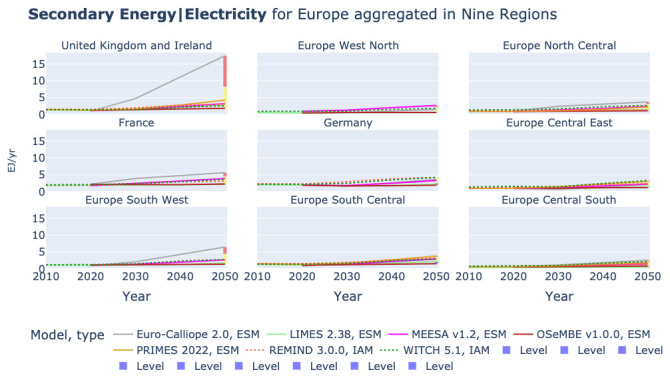
Electricity generation in nine region aggregation across models.

In Figure 6 we show the electricity generation in the nine-region aggregation. Most models also provide results in the nine-region aggregation, only IMAGE, MESSAGEix, PROMETHEUS, and TIAM-ECN drop out. The lower aggregations shows that the high values of power generation in Euro-Calliope are mainly linked to power generation in the United Kingdom and Ireland and to a limited extent Europe South West, which consists of Portugal and Spain. This indicates the relevance of the availability of regional disaggregated model results. However, it does not indicate the origin of the high electricity generation in Euro-Calliope in particular and the reasons for differences across the other models in general.

**Figure 7.**
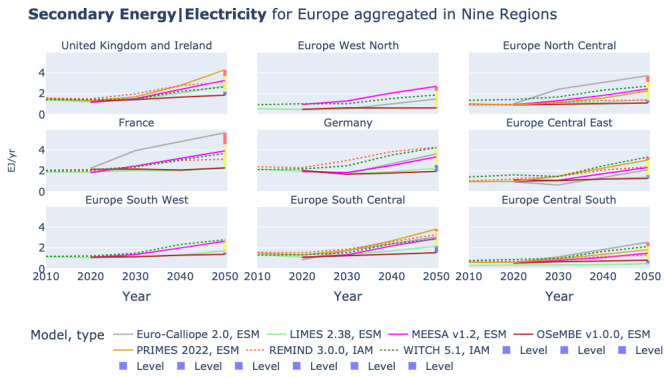
Electricity generation in nine region aggregation across models without Euro-Calliope in United Kingdom and Ireland and Europe South West.

All other models show a more even distribution of secondary electricity generation across the nine regions, as shown in Figure 7. In Figure 7 Euro-Calliope is removed from the plots for the United Kingdom and Ireland and Europe South West to improve the readability of the of the plots. The figure shows that the distribution of models varies across regions.

### Hydrogen production from electricity

**Figure 8.**
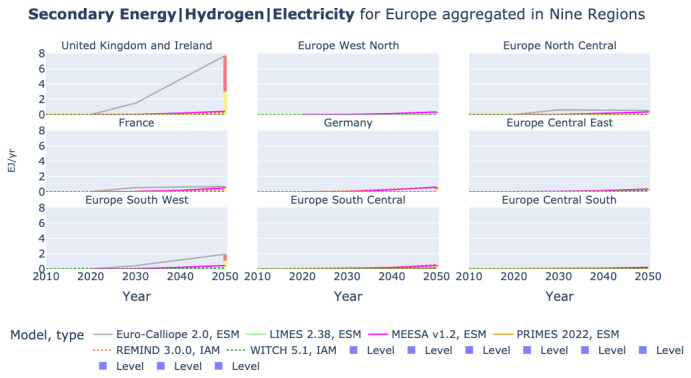
Hydrogen production from electrolysis in nine region aggregation across models.

The amount of electricity generated is related to the amount of electricity demanded across sectors, both for end-use and as mean to produce hydrogen. As an example, we show in Figure 8 the use of electricity for hydrogen production in the nine-region aggregation across models. We can note that a key driver of the high electricity generation in Euro-Calliope is the hydrogen production in the United Kingdom and Ireland, but also in Spain and Portugal.

**Figure 9.**
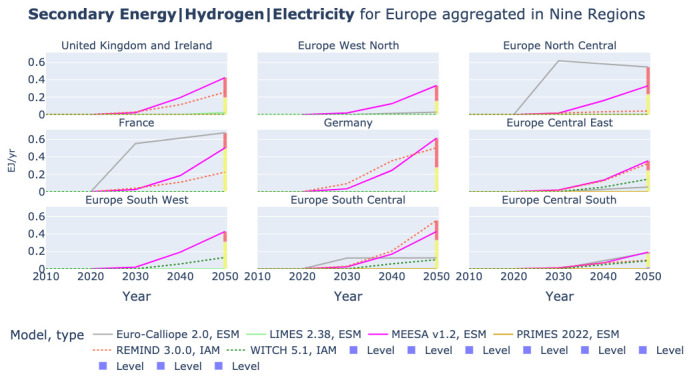
Hydrogen production from electrolysis in nine region aggregation across models without Euro-Calliope in United Kingdom and Ireland and Europe South West.

Figure 5 also shows that REMIND is the model with the second highest electricity generation. Hence, we consider it interesting to have a look at the hydrogen production across regions removing the Euro-Calliope values for the United Kingdom and Ireland and Europe South West. We show this in Figure 9. It becomes apparent that Euro-Calliope is also expecting the highest hydrogen production in Europe North Central and France. However, in the other five regions Euro-Calliope’s hydrogen production is low.

REMIND and Euro-Calliope show different spatial patterns of hydrogen production. While Euro-Calliope produces hydrogen in regions with better renewable resources, REMIND generates hydrogen more evenly across regions. Figure 9 shows that REMIND consistently produces high levels of H2 in seven of the nine regions. Only in Europe North Central and Europe Central South it is not producing highest or second highest after Euro-Calliope.

In Section 2.3 we illustrate that ESOMs do not fully report the compared hydrogen variables. This links to the fact that the models LIMES and WITCH do not cover a wide range of potential uses of hydrogen, but rather focus on the usage of hydrogen in the context of the power generation and in some case the heating sector. Furthermore, the OSeMBE model does not model hydrogen at all. The low usage of electricity for hydrogen production in LIMES and WITCH is therefore not surprising.

### Onshore wind power

**Figure 10.**
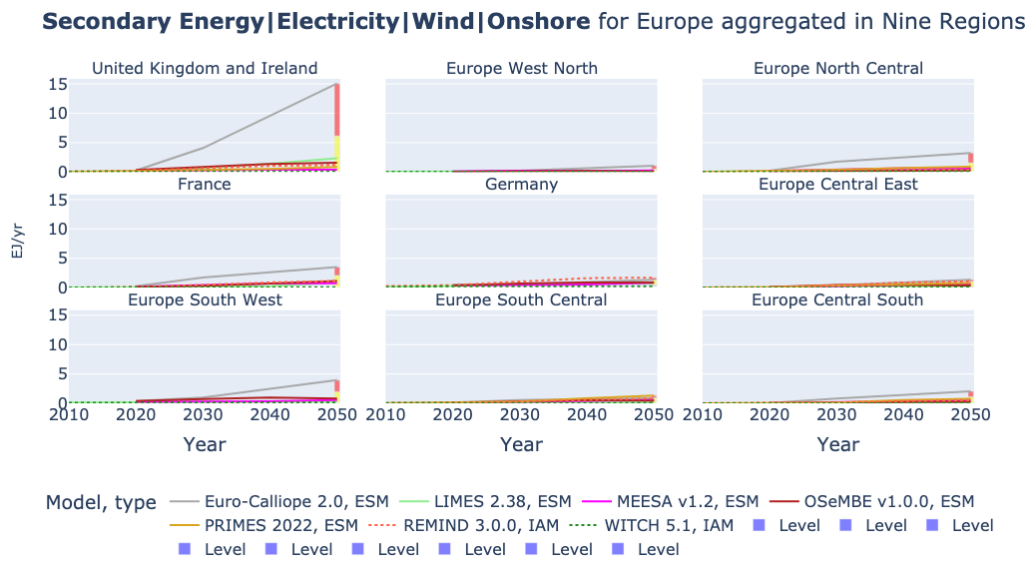
Electricity generation from Onshore Wind in nine regions across models.

Figure 10 illustrates that the high production of hydrogen in Euro-Calliope and REMIND correlates with the electricity generation from onshore wind. Like for hydrogen, Euro-Calliope produces most electricity from onshore wind in the United Kingdom and Ireland and also in Europe North Central, France, Europe South West, and Europe Central South it anticipates a higher power generation from onshore wind than all other models in the comparison. Similarly REMIND is in most regions on the high side for onshore wind power, but like for hydrogen with smaller differences across regions than Euro-Calliope.

In Table 3 we can observe that a key reason for the high deployment of onshore wind in Euro-Calliope is the resource availability assumed for onshore wind. The capacity factor in Euro-Calliope, calculated using the installed capacity and the power production, is with 38.2% high in comparison to the other models. In REMIND, additional equations representing wind and solar integration challenges favor a higher share of wind than solar in the EU, given that both electricity demand and wind generation are higher in winter.

**Table 3.** Comparison of variables in 2050 across models with color coding for high (red), medium (yellow), and low (blue)

	Model	IMAGE 3.2	MESSAGEix-GLOBIOM 1.2	PROMETHEUS 1.2	REMIND 3.0.0	TIAM-ECN 1.2	WITCH 5.1	Euro-Calliope 2.0	LIMES 2.38	MEESA v1.2	OSeMBE v1.0.0	PRIMES 2022
	Type	IAM	IAM	IAM	IAM	IAM	IAM	ESM	ESM	ESM	ESM	ESM
	Algo.	Sim	Opt	Sim	Opt	Opt	Opt	Opt	Opt	Opt	Opt	Opt
	Unit											
**Final Energy|Electricity**	EJ/yr	13	23	15	19	23	22	23	13	23	12	15
**Secondary Energy|** **Electricity**	EJ/yr	15	29	17	25	29	25	46	15	30	13	30
**Share|** **variable Renewable Energies**	%	46	59	48	88	85	73	95	83	73	58	73
**Secondary Energy|** **Electricity|Solar|PV**	EJ/yr	4	8	3	9	15	11	11	7	8	2	6
**Capacity|Electricity|** **Solar|PV**	GW	857	1537	970	2472	2190	1949	2606	1465	1790	393	1421
**Capacity Factor|Solar|PV**	%	14	17	11	12	22	18	13	14	14	14	13
**Capital Cost|Electricity|** **Solar|PV**	€_2020 /kW	392	511	609	328	352	292		351	435	517	444
**Secondary Energy|** **Electricity|Wind|Onshore**	EJ/yr	2	8	4	9	4	1	32	5	6	6	9
**Capacity|Electricity|** **Wind|Onshore**	GW	258			1009	607	206	2686	504	688	672	1036
**Capacity Factor|** **Wind|Onshore**	%	30			28	21	11	38	33	26	27	27
**Capital Cost|Electricity|** **Wind|Onshore**	€_2020 /kW	1156	986	1110	1107	880	783	960	1045	925	1230	971
**Secondary Energy|** **Electricity|Wind|Offshore**	EJ/yr	0	0	1	3	5	4	0	1	8		7
**Capacity|Electricity|** **Wind|Offshore**	GW	36			252	400	373	24	45	569		489
**Capacity Factor|** **Wind|Offshore**	%	37			43	40	36	41	46	47		48
**Capital Cost|Electricity|** **Wind|Offshore**	€_2020 /kW	1487	1478	2144	1634	1976	1966	1680	2023	2160	2668	2029
**Secondary Energy|** **Hydrogen|Electricity**	EJ/yr	0	2		3	1	0	11	0	4		0
**Capital Cost|** **Hydrogen|Electricity**	€_2020 /kW	348	700	480		360		437	530	350		416
**Efficiency|** **Hydrogen|Electricity**	%		80		40	74		72	70	70		85
**Secondary Energy|** **Electricity|Nuclear**	EJ/yr	0	3	2	1	1	2	1	0	4	1	2
**Capital Cost|** **Electricity|Nuclear**	€_2020 /kW	2235	5767	5400	7232	3942	4454	3672	7367	4500	3878	5544
**Capacity|Electricity|** **Nuclear**	GW	19	114	46	23	76	62	41	5	156	39	82

### The share of variable renewable energies and details lost due to aggregation

**Figure 11.**
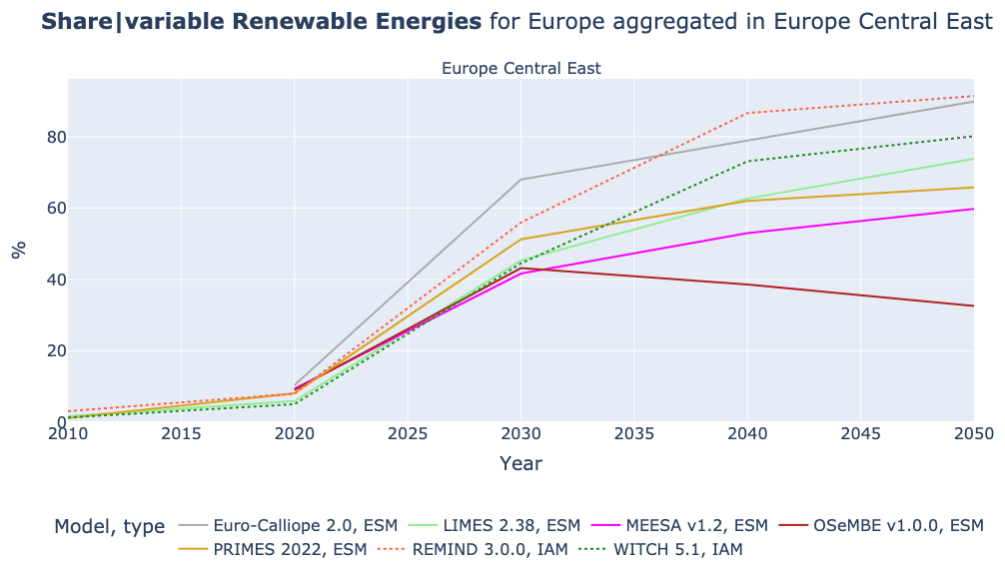
Share of variable renewable energies in Electricity generation in Europe Central East.

The comparison across models with different regional aggregations allows one to investigate aspects that are otherwise lost due to aggregation. As an example, Figure 11 shows the share of variable renewable energies in Europe Central East, which consists of Czech Republic, Estonia, Latvia, Lithuania, Poland, and Slovakia. The results show a range of values in the region, but we are not able to see whether the renewable resources are homogenously distributed within the region or if some regions will be able to reach higher shares than others.

**Figure 12.**
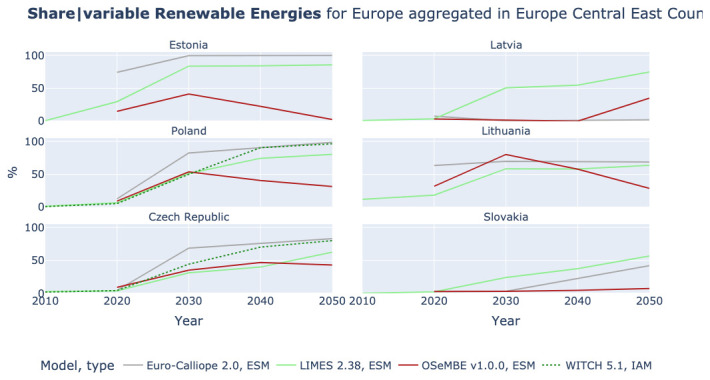
Share of variable Renewable Energies in Electricity generation in Europe Central East by country.

Figure 12 shows the share of variable renewable energies by country in Europe Central East. Even though the models do not fully align, Slovakia and Latvia have lower shares than the aggregated results in Figure 11 suggest. In contrast, Estonia and Poland reach higher shares than the aggregated results suggest. With some exceptions, scenarios from the Euro-Calliope and LIMES models agree on a stronger role for renewable generation in Estonia and Poland, a middling role in Czech Republic, Slovakia and Lithuania but disagree on the role for renewables in Latvia. However, the results do not show a broad agreement at the national scale, echoing the wide range in the aggregated 9-region results. This indicates that even though insights relating to the role of renewables at a European scale are robust, significant disagreement remains between models, representative of the uncertain implementation at national levels. This finding is not necessarily negative, as clearly there are multiple alternative pathways for the deployment of high-penetrations of renewable energy, with countries able to switch roles as indicated in Figure 12. However, it indicates that more work needs to be conducted to better harmonize or link national and European scenarios across models; and that it is important to understand the implications at a national scale of more aggregate results.

### The role of nuclear power

**Figure 13.**
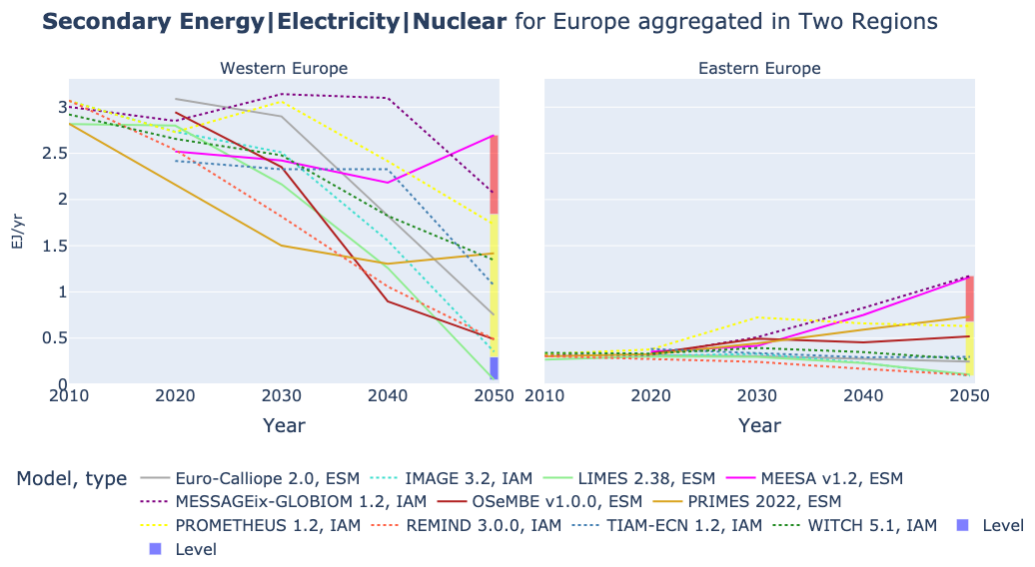
Electricity generation by Nuclear in two regions across models.

**Figure 14.**
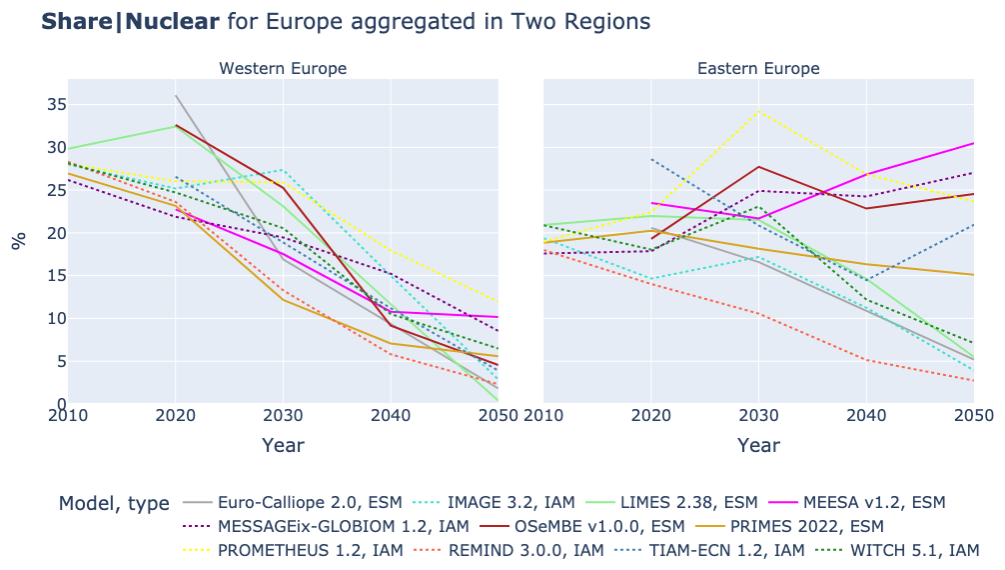
Share of Nuclear power in Electricity generation in Europe in two regions across models.

In Western Europe most models expect a decline of nuclear power, see left plot in Figure 13. However, in PRIMES and MEESA an increase can be observed that can be linked to France and the UK. In Eastern Europe the picture is more mixed, see right plot in Figure 13. Five models expect an increasing role of nuclear power, MEESA, MESSAGEix, OSeMBE, PRIMES, and PROMETHEUS. This difference between Western Europe and Eastern Europe is confirmed by Figure 14. In almost all models the shares of nuclear power in electricity generation drop to below 10% in Western Europe. However, in Eastern Europe two groups of models are observable. The five models that also expect higher absolute power generation and TIAM-ECN anticipate shares of above 15% and mostly between 20% and 30% in 2050, while the other models reduce the share of nuclear power to below 10% like in Western Europe.

Table 3 shows that the low generation of nuclear power in LIMES and REMIND might be caused by the relatively high capital cost, with REMIND substituting nuclear with onshore wind and LIMES showing low power generation and final electricity use. However, the IMAGE model assumes the lowest capital cost for nuclear power of all models in the comparison, despite the fact that it shows a lower power generation by nuclear than REMIND. A possible explanation could be the low value of Final Energy|Electricity in IMAGE.

Furthermore, the use of nuclear seems linked to the availability of wind resources. In the renewable resource rich countries France and UK that both have plans for new nuclear power most models show anyhow a decline, whereas in Eastern Europe where wind conditions are poorer, more model results exhibit an increase in power generation from nuclear. With the set-up of this study it cannot be determined whether models do not fully align due to parametric constraints or structural reasons.

## 4 Discussion

In the previous sections we first mapped out the different region aggregations across models, identified variables that are reported by both IAMs and ESOMs and presented the results comparison for some of the jointly reported variables. The variables we presented address four central aspects of decarbonization scenarios. These aspects are ‘power generation’, ‘hydrogen production’, the ‘role of variable renewables’ and the ‘role of nuclear power’.

Generally IAMs have a more aggregated approach than ESOMs – see Figure 2. The mapping of the different region aggregations showed that there are predominant model region aggregations with models of the EU energy sector aggregated into two or nine regions, but that the actual aggregation of countries often varies from model to model, highlighted in Figure 2 by the marked harmonised regions. This prevents accurate and detailed model comparison. For example, how the difference in aggregation between PRIMES and MEESA and REMIND regarding Germany and France hampers the model comparison becomes visible in Figure 6, PRIMES does not model the two countries individually. The nine-region resolution used by MEESA and REMIND shows very similar levels of final electricity demand across regions. Integrating Germany and France into other regions would distort this. These variations are an obstacle for consistent model comparisons which could be overcome with better coordination across modelling teams to use harmonised native regions, while moving beyond the EU-wide assessments and provide more disaggregated information on key decarbonisation strategies.

The variable mapping shows for the power sector that both types of models provide results at the same level of detail by energy carrier. However, ESOMs could provide more detailed results.

For the demand side, heat, and hydrogen, the mapping shows that the compared ESOMs seem to be more aggregated in comparison to the involved IAMs – see Table 2. But, at least in the case of Euro-Calliope, this is not the case. Euro-Calliope represents heat with a high level of detail and distinguishes between different technological supply options such as district heating vs. stand-alone. However, it does not distinguish between economic sectors. Therefore, it reports only one type of heat, when using the IAMC-nomenclature.

Nevertheless, for ESOMs with a limited sectoral coverage, hydrogen demands derived from IAMs and full system ESOMs could be a variable that could be used as an input. Another option for information flow between models could be the above-mentioned sectoral demands from IAMs and full system models. But, as we noticed for the case of heat in Euro-Calliope, the issue here might not be that the models are not modelling certain demands with more detail but rather with different detail, e.g., instead of by economic sector with higher technological resolution. In such a case a model linking might be difficult, but potentially a comparison of the different representations would bring benefits for both model types. ESOMs could refine their representation knowing about economic sectors, and IAMs could refine their representation of technological detail.

In the results section we show that the compared models show a wide range of expected power generation. The differences in power generation link to differences in electricity demand levels which possibly link to the expected levels of electrification. In this context we illustrate the example of hydrogen production by electrolysis and can show that the high electricity generation coincides with high generation of hydrogen. The hydrogen production correlates with the power generation from onshore wind, but differences across models are observable regarding the geographic distribution of hydrogen production and onshore wind generation.

We also manage to illustrate how high regional aggregation can conceal regional differences on the example of expected shares of variable renewable energies in the power mix. This could for example be an obstacle for governments, particularly of smaller countries, when using the results of IAMs.

The results in Table 3 also show that the two simulation models in this comparison, IMAGE and PROMETHEUS, are among the models with lower final electricity usage and lower electricity generation. Furthermore, both models show relatively low shares of variable renewable energies, despite that the low levels of power generation should facilitate higher shares.

Lastly, we investigate the role of nuclear power across models and regions. We can observe two main patterns. Firstly, the deployment shows correlation with the availability of wind resources, in Western Europe a declining trend of power generation from nuclear can be observed while in Eastern Europe, where wind resources are more limited than in Western Europe, we observe a mixed picture.

## 5 Conclusions

In conclusion the work conducted for this paper highlights the following:

- Despite region aggregations with similar number of regions, IAMs and ESOMs differ in the aggregation of countries to regions, which hampers direct model comparison.
- A model comparison of a wide range of variables across different regional aggregations can identify and trace differences in results between models to their origin.
- Variable mapping can facilitate the identification of commonly reported variables and can thereby ease model comparisons. It also facilitates the identification of possible information flows between models of different sectoral coverage.

Common standards for region aggregation could facilitate model comparison exercises. Identifying harmonised regions through a mapping exercise, as conducted for this paper, can help lead to a more effective comparison of results. We highlight two levels of region aggregation across which ESOMs and IAMs can be compared. The two-region level is the most aggregate and allows the comparison of all models in the comparison. But removes some of the detailed insights from the ESOMs. The nine-region level provides a greater opportunity for comparison with ESOMs, because it allows a better consideration of regional differences in resource availability and demand, while reducing the computational effort that comes along with a country resolution. However, the varying region aggregations highlighted by the attempt to define harmonised regions in Figure 2 represent an obstacle for detailed model comparisons. A potential approach in future to define harmonised regions could involve optimisation techniques. This would allow to systematically consider different dimensions of the decision on how to group countries to regions.

The mapping of reported variables is a simple analysis of the data reported by models in a model comparison. But simple as it might be, it facilitates the usage of the reported data for analysis and facilitates the later addition of other models to the comparison by giving an overview of what the most common variables reported are. Therefore, a conclusion of this paper is that platforms such as the IIASA Scenario Explorer – that has been used for the work presented here – could increase the likelihood that their database will be further used and expanded after initial project funding has ended by providing statistics on how many models have reported a variable. This allows modelling teams that are adding their results later and that are perhaps not even part of the initial project to better identify what are the core variables to report. Policy makers would also obtain a better understanding of what insights models do deliver and how well that aligns with what they consider relevant.

The variable comparison highlights that the sectoral coverage of the compared IAMs and ESOMs differs, but also that there is an overlap in reported variables. It also highlights that the IAMC-nomenclature could be expanded to allow a better consideration of the differences in modelling techniques between IAMs and ESOMs, which in turn would allow more in-depth comparison.

The presented region mapping of models for the EU and UK is a novel addition to the literature by providing insights in how models define regions differently.

The results analysis shows how correlations between variables can be identified and thereby allow tracing the sources of differences across models. The analysis also highlights how in the compared complex models observed effects are commonly not monocausal. The comparison across different aggregations shows that the models differ to a greater degree than the comparison of the aggregated European variables indicates. Comparing models at lower aggregation shows that the distribution of technology deployment varies between models. Lastly, the comparison illustrates that there are parametric causes for the observed differences across models. However, structural reasons cannot be ruled out.

The comparison of IAMs commonly focuses on the EU or even global level. The here presented disaggregation provides more detailed modelling results for a decarbonisation scenario for regions within the EU. The region mapping and variable mapping together highlight that for standardised model comparisons and potential model linking a better harmonisation of region aggregations and information on commonly reported variables and their meaning is required. This underlines the relevance of the ECEMF project and its objective of providing an open-source full scale model comparison to the European modelling community.

## Data Availability

The data underlying this study is available at: https://data.ece.iiasa.ac.at/ecemf Zenodo: ECEMF Diagnostic Scenarios, version 3.0 https://zenodo.org/doi/10.5281/zenodo.7634844 ( Henke, 2023a) This project contains the following underlying data: ECEMF-diagnostic-scenarios-v1.xlsx (Archived underlying data at time of publication. Zenodo: Interactive plots for all variables identified in Table 2 that are reported by more than two models, at three resolutions. https://doi.org/10.5281/zenodo.10797680 ( Henke, 2023b). Data are available under the terms of the Creative Commons Attribution 4.0 International license (CC-BY 4.0). Repository: https://github.com/HauHe/ESMsxIAMs_figs/tree/v0.2.0 Webpage: https://hauhe.github.io/ESMsxIAMs_figs/ List of variables with available plots: **Emissions** • CO2|Energy|Supply|Electricity • CH4|Energy|Supply **Demands** (Final Energy) • Final Energy • Electricity • Residential and Commercial • Residential and Commercial|Electricity • Commercial • Commercial|Electricity • Residential • Residential|Electricity • Transportation • Transportation|Electricity **Primary Energy** • Biomass|Electricity • Coal|Electricity • Gas|Electricity • Oil|Electricity **Electricity Supply** (Secondary Energy|Electricity) • Biomass • Coal • Gas • Geothermal • Hydro • Nuclear • Ocean • Oil • Solar • Solar|CSP • Solar|PV • Wind • Wind|Offshore • Wind|Onshore **Heat** • Final Energy|Heat • Secondary Energy|Heat **Hydrogen** • Final Energy|Hydrogen • Secondary Energy|Hydrogen • Secondary Energy|Hydrogen|Electricity

## Software availability

Source code available from: https://github.com/HauHe/ESMsxIAMs/tree/v1.0.1

Archived source code at time of publication: https://doi.org/10.5281/zenodo.10797690 ( Henke, 2023a)

License: MIT
