# Supplementary material for: Comparing energy system optimization models and integrated assessment models: Relevance for energy policy advice
Source: Open Res Eur. 2024 Mar 21;3:69. Originally published 2023 May 2. [Version 2] doi: 10.12688/openreseurope.15590.2 (PMC11043660; doi:10.12688/openreseurope.15590.2)
Supplement: Peer review history

# Reviewer report: Reviewer response for version 2

**DOI:** 10.21956/openreseurope.18896.r39071

**Published:** 2024-04-24

**Authors:** Jesse Jenkins

**Affiliations:** 1 Andlinger Center for Energy and the Environment, Princeton University, Princeton, New Jersey, USA

The revisions to this paper tackle the key concerns shared in my initial review. I commend the authors for taking this constructive criticism to heart. The resulting paper is much improved and I believe now offers a compelling contribution to the field.

Is the study design appropriate and does the work have academic merit?

Partly

Is the work clearly and accurately presented and does it cite the current literature?

Partly

If applicable, is the statistical analysis and its interpretation appropriate?

Not applicable

Are all the source data underlying the results available to ensure full reproducibility?

Yes

Are the conclusions drawn adequately supported by the results?

Partly

Are sufficient details of methods and analysis provided to allow replication by others?

Yes

Reviewer Expertise:

Macro-energy systems. Energy systems models. Capacity expansion models. Decarbonisation. Energy transitions. Energy technology. Energy policy. Optimization. Electricity Regulation.

I confirm that I have read this submission and believe that I have an appropriate level of expertise to confirm that it is of an acceptable scientific standard.

# Reviewer report: Reviewer response for version 2

**DOI:** 10.21956/openreseurope.18896.r39070

**Published:** 2024-04-12

**Authors:** David McCollum

**Affiliations:** 1 Oak Ridge National Laboratory, Oak Ridge, Tennessee, USA

The authors have done a solid job of reworking their manuscript, taking my and the other reviewers' feedback into account.

Is the study design appropriate and does the work have academic merit?

Yes

Is the work clearly and accurately presented and does it cite the current literature?

Yes

If applicable, is the statistical analysis and its interpretation appropriate?

Yes

Are all the source data underlying the results available to ensure full reproducibility?

Partly

Are the conclusions drawn adequately supported by the results?

Partly

Are sufficient details of methods and analysis provided to allow replication by others?

Partly

Reviewer Expertise:

Integrated Assessment Modeling, Energy Systems Modeling

I confirm that I have read this submission and believe that I have an appropriate level of expertise to confirm that it is of an acceptable scientific standard.

# Reviewer report: Reviewer response for version 1

**DOI:** 10.21956/openreseurope.16850.r31913

**Published:** 2023-07-17

**Authors:** Jesse Jenkins

**Affiliations:** 1 Andlinger Center for Energy and the Environment, Princeton University, Princeton, New Jersey, USA

This paper compares results from a set of IAMs and ESMs to determine how comparable results are and to try to describe the overlaps between IAMs and ESMs the context of modelling possible European decarbonisation pathways and how these overlaps might vary depending on the model implementation.

 These kinds of exercises can be worthwhile, and I was very intrigued by the premise, to see how these two classes of commonly used macro-energy systems models compares and what drivers their differences.

 However, the paper's results section is basically entirely descriptive, with no analysis of what drives the substantial differences in results across the models. Without such discussion, this paper does not offer a whole lot beyond a catalog of results, which could be shared as a data set. To be a useful contribution to the field, a more thorough focus on WHY the results are as they are is needed, not simply description of the observed results. Without such analysis, I cannot recommend the current manuscript for indexing.

 Suggestion:

 Try to visually distinguish IAMs and ESMs in all plots. e.g. solid lines for IAMs dotted for ESMs or something similar. This is a key distinction in the models and difficult to contrast visually in your results unless one remembers which of the arcane abbreviations corresponds to which type of model.

 Notes:

 "Energy system models (ESMs), instead, provide in-depth and context-specific insights on the technological transition required to decarbonize the energy system with commonly more detailed representation of temporal and spatial details." - ESMs also typically have a richer and more detailed representation of technological options for decarbonization, such as more detailed operational constraints or a wide range of options for decarbonization in specific sectors.

 "The differences can be structural, i.e., in the set-up of the modelling framework, or parametric, which means related to the input data, the selected value of model parameters, and to the boundary conditions." - Another way I like to explain the difference between structural and parametric differences is that structural decisions relate to HOW the model represents the world and parametric differences relate to WHAT state of the world the model is trying to represent. May be helpful to explain that way for broader audience (just a suggestion).

 "Understanding the differences between models improves the understanding **u** of whether the insights derived from the models are robust or not." Type there in bold.

 "The fields of integrated assessment modelling and energy systems modelling contain subjective assumptions to a higher degree than climate models." -  The key difference is not so much that they contain subjective assumptions, but rather than these models represent *socio*-technical systems not purely physical systems. As such, they don't simply behave based on rules of physics, but also society (economics, policy, behavior) which are far more difficult to reliably represent in models. This prompts a larger diversity of structural choices in model development and use, and it challenges model validation efforts.

 RESULTS Section:

 3.1 Demands: The final energy demands and final energy electricity are quite different across models, but this goes without comment. The discussion focuses on whether demands are increasing or decreasing, but does not note the large difference in levels seen in Fig 5.

Is the study design appropriate and does the work have academic merit?

Partly

Is the work clearly and accurately presented and does it cite the current literature?

Partly

If applicable, is the statistical analysis and its interpretation appropriate?

Not applicable

Are all the source data underlying the results available to ensure full reproducibility?

Yes

Are the conclusions drawn adequately supported by the results?

Partly

Are sufficient details of methods and analysis provided to allow replication by others?

Yes

Reviewer Expertise:

Macro-energy systems. Energy systems models. Capacity expansion models. Decarbonisation. Energy transitions. Energy technology. Energy policy. Optimization. Electricity Regulation.

I confirm that I have read this submission and believe that I have an appropriate level of expertise to state that I do not consider it to be of an acceptable scientific standard, for reasons outlined above.

# Response

**Published:** 2024-03-07

**Authors:** Hauke Henke

**Affiliations:** KTH Royal Institute of Technology, Sweden

Dear Jesse,

 Thank you for providing your review for our paper on the comparison of Integrated Assessment Models and Energy System Optimisation Models. In the following we provide a point-by-point response to your comments.  

*JJ: ‘* *However, the paper's results section is basically entirely descriptive, with no analysis of what drives the substantial differences in results across the models. Without such discussion, this paper does not offer a whole lot beyond a catalog of results, which could be shared as a data set. To be a useful contribution to the field, a more thorough focus on WHY the results are as they are is needed, not simply description of the observed results.’*  

**Authors: **This is a valid point. We have worked on providing more depth to our analysis and shed light on the relations and reasons for the observed dynamics in the results. In the new results analysis, the focus is put on a set of results that is well covered by the compared models and that is of high policy relevance. The selected set of results has five foci: final electricity demand, hydrogen production via electrolysis, onshore wind power, renewable energies in power production, and nuclear power. In the analysis we show how these five topics are interrelated and identify causes for the differences between the compared models. Furthermore, we illustrate how the high regional aggregation used in many of the compared models can hide regional heterogeneity and consequentially limit the usability of model results for national policy makers. Drawing from the insights provided by the reworked results section, we have also updated our discussion and conclusion sections, where we are now able to better highlight the benefits and inherent challenges of comparing IAMs and ESMs. We believe the changes made increase the depth of the paper and we hope they will find your approval.  

*JJ: ‘* *Try to visually distinguish IAMs and ESMs in all plots. e.g. solid lines for IAMs dotted for ESMs or something similar. This is a key distinction in the models and difficult to contrast visually in your results unless one remembers which of the arcane abbreviations corresponds to which type of model.’*   Authors: Thanks for this tip. We are now illustrating ESMs with solid lines and IAMs with dotted lines. *JJ: ‘* *Energy system models (ESMs), instead, provide in-depth and context-specific insights on the technological transition required to decarbonize the energy system with commonly more detailed representation of temporal and spatial details." - ESMs also typically have a richer and more detailed representation of technological options for decarbonization, such as more detailed operational constraints or a wide range of options for decarbonization in specific sectors.’*  

**Authors:** Thank you for this comment, we expanded our sentence.  

*JJ: ‘* *"The differences can be structural, i.e., in the set-up of the modelling framework, or parametric, which means related to the input data, the selected value of model parameters, and to the boundary conditions." - Another way I like to explain the difference between structural and parametric differences is that structural decisions relate to HOW the model represents the world and parametric differences relate to WHAT state of the world the model is trying to represent. May be helpful to explain that way for broader audience (just a suggestion).’*  

**Authors:** Nice clarification. We adjusted our phrasing in the corresponding section of the introduction.  

*JJ: ‘* *"Understanding the differences between models improves the understanding u of whether the insights derived from the models are robust or not." Type there in bold.’*  

**Authors:** We set the font to bold and removed the typo 'u'.  

*JJ: ‘* *"The fields of integrated assessment modelling and energy systems modelling contain subjective assumptions to a higher degree than climate models." - The key difference is not so much that they contain subjective assumptions, but rather than these models represent socio-technical systems not purely physical systems. As such, they don't simply behave based on rules of physics, but also society (economics, policy, behavior) which are far more difficult to reliably represent in models. This prompts a larger diversity of structural choices in model development and use, and it challenges model validation efforts.’*  

**Authors: **Thanks a lot for this guiding comment. We expanded our text on this, see third paragraph in the introduction.  

*JJ: ‘* *3.1 Demands: The final energy demands and final energy electricity are quite different across models, but this goes without comment. The discussion focuses on whether demands are increasing or decreasing, but does not note the large difference in levels seen in Fig 5.’*

**Authors: **Thanks for pointing this out. As part of the restructuring of the results section we have decided to put our focus more on the power generation, since this more in line with the focus of the ESOMs in the comparison. However, in the comparison of power generation (Secondary Energy|Electricity) across models we also observe a wide spread among the models. We therefore also investigate the hydrogen production from electrolysis one potential driver for electricity production.

# Reviewer report: Reviewer response for version 1

**DOI:** 10.21956/openreseurope.16850.r31914

**Published:** 2023-07-17

**Authors:** David McCollum

**Affiliations:** 1 Oak Ridge National Laboratory, Oak Ridge, Tennessee, USA

This paper reports findings and insights from a model inter-comparison between a heterogenous mix of models. Specifically, integrated assessment models (IAMs) and energy systems models (ESMs) are compared and contrasted – something that is rarely done, given the overlapping yet separate nature of the corresponding research communities. In this sense, the mere act of undertaking the project is a success in and of itself. Certainly, this can be counted as one achievement of the European Climate and Energy Modelling Forum.

 The manuscript is solid – well written and educational. It will surely become a key reference in the modeling field (multiple communities) going forward, and not just for European-focused researchers.

 That being said, I do wonder whether the authors have peeled back the layers of the onions enough. It could be that their insights are in some ways pre-determined by their methodology.

 Some specific comments below…

 P. 3 (of the pdf version of the article), top-left => I would strongly suggest avoiding using the 'ESM' term here, given that it is more commonly associated with 'Earth System Models', particularly in a model inter-comparison context (think CMIP, etc.).

 P. 8, bottom-left => The insights on demand-side detail and hydrogen and heat coverage are in some ways unexpected and counter-intuitive; they deserve a bit more explanation. One would expect ESMs to have greater detail. I do note the sentences of explanation in the first paragraph on p. 10. This is a start.

 P. 11, graphics => Here and elsewhere the authors may want to add a few sentences explaining why there are significant differences in energy demand and supply in 2020 (a historical year at this point, but possibly a projected year in some models).

 P. 18, bottom-left => Regarding variable mapping, I would think that the energy systems models would have greater detail. It's just that certain variables at higher resolution were not originally listed in the IAMC reporting template, and thus are outside the scope of this analysis. More generally, by starting from the IAMC reporting template, some findings could be in a way pre-determined. I note one of the conclusions mentioned on p. 20 (bottom-left), which gives a nod in this direction.

 P. 18, bottom-left => Typo: IMAC should be IAMC.

 P. 18, top-right => It would be insightful to know whether there are differences between models with different solution algorithms (simulation vs. optimization), no matter whether the model is an IAM or ESM. That could be telling.

Is the study design appropriate and does the work have academic merit?

Yes

Is the work clearly and accurately presented and does it cite the current literature?

Yes

If applicable, is the statistical analysis and its interpretation appropriate?

Yes

Are all the source data underlying the results available to ensure full reproducibility?

Partly

Are the conclusions drawn adequately supported by the results?

Partly

Are sufficient details of methods and analysis provided to allow replication by others?

Partly

Reviewer Expertise:

Integrated Assessment Modeling, Energy Systems Modeling

I confirm that I have read this submission and believe that I have an appropriate level of expertise to confirm that it is of an acceptable scientific standard, however I have significant reservations, as outlined above.

# Response

**Published:** 2024-03-07

**Authors:** Hauke Henke

**Affiliations:** KTH Royal Institute of Technology, Sweden

Dear David,

 Thank you for providing your review for our paper on the comparison of Integrated Assessment Models and Energy System Optimisation Models. In the following we provide a point-by-point response to your comments.  

*DMC: ‘* *That being said, I do wonder whether the authors have peeled back the layers of the onions enough. It could be that their insights are in some ways pre-determined by their methodology.’*  

**Authors: **This is a valid point. We have worked on providing more depth to our analysis and shed light on the relations and reasons for the observed dynamics in the results. In the new results analysis, the focus is put on a set of results that is well covered by the compared models and that is of high policy relevance. The selected set of results has five foci: final electricity demand, hydrogen production via electrolysis, onshore wind power, renewable energies in power production, and nuclear power. In the analysis we show how these five topics are interrelated and identify causes for the differences between the compared models. Furthermore, we illustrate how the high regional aggregation used in many of the compared models can hide regional heterogeneity and consequentially limit the usability of model results for national policy makers. Drawing from the insights provided by the reworked results section, we have also updated our discussion and conclusion sections, where we are now able to better highlight the benefits and inherent challenges of comparing IAMs and ESMs. We believe the changes made increase the depth of the paper and we hope they will find your approval.  

*DMC: ‘* *P. 3 (of the pdf version of the article), top-left => I would strongly suggest avoiding using the 'ESM' term here, given that it is more commonly associated with 'Earth System Models', particularly in a model inter-comparison context (think CMIP, etc.).’*  

**Authors:** Thanks for the comment. We are now using the abbreviation ESOM for Energy System Optimization Model.  

*DMC: ‘* *P. 8, bottom-left => The insights on demand-side detail and hydrogen and heat coverage are in some ways unexpected and counter-intuitive; they deserve a bit more explanation. One would expect ESMs to have greater detail. I do note the sentences of explanation in the first paragraph on p. 10. This is a start.’*  

**Authors: **Thanks for this comment. We have changed the formulation to clarify that we are illustrating mainly a reporting issue and only to a limited extend limitations by the models.  

*DMC: ‘* *P. 11, graphics => Here and elsewhere the authors may want to add a few sentences explaining why there are significant differences in energy demand and supply in 2020 (a historical year at this point, but possibly a projected year in some models).’*  

**Authors: **Thank you for pointing this out. We have worked on this aspect and compared the model results with statistical numbers from Eurostat and adjusted the models to the extent possible to capture the historic developments. We clarify this also in the paper at the end of section 2.1.  

*DMC: ‘* *P. 18, bottom-left => Regarding variable mapping, I would think that the energy systems models would have greater detail. It's just that certain variables at higher resolution were not originally listed in the IAMC reporting template, and thus are outside the scope of this analysis. More generally, by starting from the IAMC reporting template, some findings could be in a way predetermined. I note one of the conclusions mentioned on p. 20 (bottom-left), which gives a nod in this direction.’*  

**Authors:** Thanks for the comment. This is correct and we highlight this now in the discussion.

*DMC: ‘* *P. 18, bottom-left => Typo: IMAC should be IAMC.’*  

**Authors: **Thanks for spotting this typo.  

*DMC: ‘* *P. 18, top-right => It would be insightful to know whether there are differences between models with different solution algorithms (simulation vs. optimization), no matter whether the model is an IAM or ESM. That could be telling.’*  

**Authors: **Thanks for this question. We are now indicating in our new variable comparison table wheter the models are simulation or optimisation models and discuss the related observations in the discussion section. Only two of the compared models are simulation models. Therefore, we can’t draw general conclusions, but we can observe that these two models stick out by indicating relatively low final electricity demand and at the same time low share of variable renewable energies in the power generation mix.

# Reviewer report: Reviewer response for version 1

**DOI:** 10.21956/openreseurope.16850.r31946

**Published:** 2023-06-12

**Authors:** Paul Deane

**Affiliations:** 1 University College Cork, Cork, County Cork, Ireland

This paper compares aggregations of sets of energy/climate variables for harmonised regions across Europe for a deep decarbonization future. The authors make a good case for the need for standards for region definitions and information about model variables to benefit future comparisons from different model types.

 The methodology presented is focussed on harmonising and comparing high levels results from ESM and IAM. The paper helpfully compares results in terms of trends and projections however the study would benefit significantly from a stronger explanation of the key drivers of differences between results.  This would make clear the benefits of comparing energy system models and integrated assessment models.

**The Results and Conclusion** section of the Abstract are vague and nonspecific. I appreciate that these are short sections, but it would be helpful to present the reader some firm findings and more solid conclusions.

 Please elaborate on the sentence “The fields of integrated assessment modelling and energy systems modelling contain subjective assumptions to a higher degree than climate models” as this seems to be an important point.

 The paper states: “There has not been an alignment of inputs across the models in the ECEMF project, but a review of their results when the same carbon price trajectory is imposed and in some cases updates of input data”. Can this be explained better to a reader who is not familiar with the ECEMF project…are you assuming the same carbon price projection for all runs that are presented or just for some of them? Also, what does it mean that in some case the input data was updated?

 Section 3.1 Results

 The text section does a good job of explaining the general trends (which are clear from looking at the graphs) but the text does not sufficiently explain or communicate the drivers of differences in trends. Taking the case of Final Energy-way are results so different? Is it GDP, Population, Policy…..What is the insight here for the reader? What is the value added of the methodology? These are not clear from the text presented. It is important to articulate the differences in Final Energy figures in detail as it impacts many of the other outputs.

 This is an issue also for the solar section 3.2.1 …what are the drivers of results, it is costs, available land area, grid infrastructure, carbon trajectories etc? The paper would greatly benefit from these explanations and it would justify the usefulness of a comparison in the first place.

 The issue is elaborated a little better in the wind results section.3.2.2 but it needs to be more definite so the reader can understand why the results are different. The results in Figure 9 are very different for (for example) Euro-Calliope 2.0 and WITCH 5.1 for the same carbon price. What is driving this? The paper states “But this possibly indicates different modelling of wind resource limits: use of electricity for hydrogen production”.  This would benefit from being firmer.

 Section 3.9 - Previously in the text it mentioned that “It is also notable that most ESMs do not fully cover hydrogen and heat, which is a challenge when investigating the synergies offered by sector coupling and possible scenarios arising from electrification, e.g., increasing use of heat pumps for heat generation.” How is this accounted for in this section? Are we only seeing a small portion of H2 potential due to model limitations?

 Once the above comments have been addressed both the Discussion and Conclusion would benefit from more detail.

 In short. The paper presents a good methodology but does not make a sufficient case in its current form as to what the value and benefit of this methodology are.

Is the study design appropriate and does the work have academic merit?

Partly

Is the work clearly and accurately presented and does it cite the current literature?

Yes

If applicable, is the statistical analysis and its interpretation appropriate?

Not applicable

Are all the source data underlying the results available to ensure full reproducibility?

Partly

Are the conclusions drawn adequately supported by the results?

No

Are sufficient details of methods and analysis provided to allow replication by others?

Partly

Reviewer Expertise:

Energy System Modelling

I confirm that I have read this submission and believe that I have an appropriate level of expertise to state that I do not consider it to be of an acceptable scientific standard, for reasons outlined above.

# Response

**Published:** 2024-03-07

**Authors:** Hauke Henke

**Affiliations:** KTH Royal Institute of Technology, Sweden

Dear Paul,

 Thank you for providing your review for our paper on the comparison of Integrated Assessment Models and Energy System Optimisation Models. In the following we provide a point-by-point response to your comments.  

*PD: ‘The paper helpfully compares results in terms of trends and projections however the study would benefit significantly from a stronger explanation of the key drivers of differences between results. This would make clear the benefits of comparing energy system models and integrated assessment models.’*  

**Authors: **This is a valid point. We have worked on providing more depth to our analysis and shed light on the relations and reasons for the observed dynamics in the results. In the new results analysis, the focus is put on a set of results that is well covered by the compared models and that is of high policy relevance. The selected set of results has five foci: final electricity demand, hydrogen production via electrolysis, onshore wind power, renewable energies in power production, and nuclear power. In the analysis we show how these five topics are interrelated and identify causes for the differences between the compared models. Furthermore, we illustrate how the high regional aggregation used in many of the compared models can hide regional heterogeneity and consequentially limit the usability of model results for national policy makers. Drawing from the insights provided by the reworked results section, we have also updated our discussion and conclusion sections, where we are now able to better highlight the benefits and inherent challenges of comparing IAMs and ESMs. We believe the changes made increase the depth of the paper and we hope they will find your approval.  

*PD: ‘* *The Results and Conclusion section of the Abstract are vague and nonspecific. I appreciate that these are short sections, but it would be helpful to present the reader some firm findings and more solid conclusions.’*  

**Authors:** Thanks for this comment. In context of updating our results and discussion section we also updated the corresponding sections in the abstract and highlight how the results show that key energy topics are interlinked.  

*PD: ‘* *Please elaborate on the sentence “The fields of integrated assessment modelling and energy systems modelling contain subjective assumptions to a higher degree than climate models” as this seems to be an important point.’*  

**Authors:** Thanks for highlighting this point. Also in response to your colleagues comment, we have expanded our explanation on this. With this point we want to highlight that IAMs and ESMs represent socio-technical and socio-economic systems that do not purely rely on the laws of physic and are hence more difficult to validate.  

*PD: ‘* *The paper states: “There has not been an alignment of inputs across the models in the ECEMF project, but a review of their results when the same carbon price trajectory is imposed and in some cases updates of input data”. Can this be explained better to a reader who is not familiar with the ECEMF project…are you assuming the same carbon price projection for all runs that are presented or just for some of them? Also, what does it mean that in some case the input data was updated?’*  

**Authors:** Thanks for these questions. The sentence that you refer to is not part of the results section anymore. However, the last paragraph in section 2.1 contains information on the ECEMF diagnostic scenarios in general and the scenario used in specific. The diagnostic scenarios are a set of scenarios designed to test model behavior under extreme conditions. The scenario we selected for this study has a high CO2 price trajectory and hence forces the model to strong decarbonization, in line with policy goals in Europe.  

*PD: ‘* *Section 3.1 Results*

*The text section does a good job of explaining the general trends (which are clear from looking at the graphs) but the text does not sufficiently explain or communicate the drivers of differences in trends. Taking the case of Final Energy-way are results so different? Is it GDP, Population, Policy…..What is the insight here for the reader? What is the value added of the methodology? These are not clear from the text presented. It is important to articulate the differences in Final Energy figures in detail as it impacts many of the other outputs.’*  

**Authors:** Thanks for this comment. We have reworked the entire results section and believe that we are now better illustrating the dynamics in the compared models. We changed the variables compared to better account for the strength of the models compared and to better illustrate the relation between variables. In section 2.1 we also indicate to what extent the models have been harmonized for the comparison.  

*PD: ‘* *This is an issue also for the solar section 3.2.1 …what are the drivers of results, it is costs, available land area, grid infrastructure, carbon trajectories etc? The paper would greatly benefit from these explanations and it would justify the usefulness of a comparison in the first place.’*  

**Authors:** Please see our reply to your previous comment.  

*PD: ‘* *The issue is elaborated a little better in the wind results section.3.2.2 but it needs to be more definite so the reader can understand why the results are different. The results in Figure 9 are very different for (for example) Euro-Calliope 2.0 and WITCH 5.1 for the same carbon price. What is driving this? The paper states “But this possibly indicates different modelling of wind resource limits: use of electricity for hydrogen production”. This would benefit from being firmer.’*  

**Authors:** Please see our reply to your previous comment.  

*PD: ‘* *Section 3.9 - Previously in the text it mentioned that “It is also notable that most ESMs do not fully cover hydrogen and heat, which is a challenge when investigating the synergies offered by sector coupling and possible scenarios arising from electrification, e.g., increasing use of heat pumps for heat generation.” How is this accounted for in this section? Are we only seeing a small portion of H2 potential due to model limitations?’*  

**Authors:** Thanks for connecting the dots on this. We have addressed this comment. In the new results section on hydrogen we comment on which models are therefore showing lower hydrogen production.  

*PD: ‘* *Once the above comments have been addressed both the Discussion and Conclusion would benefit from more detail.’*  

**Authors:** Using the insights gained from our updated results section we have expanded the discussion and conclusion. We highlight e.g. that onshore wind and hydrogen production from onshore wind coincide and that regional aggregation can hide region internal heterogeneity.  

*PD: ‘* *In short. The paper presents a good methodology but does not make a sufficient case in its current form as to what the value and benefit of this methodology are.’*  

**Authors:** Thank you for this assessment. We hope that our revised article, including an improved results section and discussion address the issues you raised.
